# UNISELF: A unified network with instance normalization and self-ensembled lesion fusion for multiple sclerosis lesion segmentation

**DOI:** 10.1016/j.media.2026.103954

**Published:** 2026-01-20

**Authors:** Jinwei Zhang, Lianrui Zuo, Blake E. Dewey, Samuel W. Remedios, Yihao Liu, Savannah P. Hays, Dzung L. Pham, Ellen M. Mowry, Scott D. Newsome, Peter A. Calabresi, Shiv Saidha, Aaron Carass, Jerry L. Prince

**Affiliations:** a Department of Electrical and Computer Engineering, Johns Hopkins University, Baltimore, MD, 21218, USA; b Department of Electrical and Computer Engineering, Vanderbilt University, Nashville, TN, 37215, USA; c Department of Neurology, Johns Hopkins School of Medicine, Baltimore, MD, 21287, USA; d Department of Computer Science, Johns Hopkins University, Baltimore, MD, 21218, USA; e Department of Radiology, Uniformed Services University of the Health Sciences, Bethesda, MD, 20814, USA

**Keywords:** 41A05, 41A10, 65D05, 65D17, Self-ensemble, Instance normalization, Domain generalization, Multiple sclerosis, Lesion segmentation

## Abstract

Automated segmentation of multiple sclerosis (MS) lesions using multicontrast magnetic resonance (MR) images improves efficiency and reproducibility compared to manual delineation, with deep learning (DL) methods achieving state-of-the-art performance. However, these DL-based methods have yet to simultaneously optimize in-domain accuracy and out-of-domain generalization when trained on a single source with limited data, or their performance has been unsatisfactory. To fill this gap, we propose a method called UNISELF, which achieves high accuracy within a single training domain while demonstrating strong generalizability across multiple out-of-domain test datasets. UNISELF employs a novel test-time self-ensembled lesion fusion to improve segmentation accuracy, and leverages test-time instance normalization (TTIN) of latent features to address domain shifts and missing input contrasts. Trained on the ISBI 2015 longitudinal MS segmentation challenge training dataset, UNISELF ranks among the best-performing methods on the challenge test dataset. Additionally, UNISELF outperforms all benchmark methods trained on the same ISBI training data across diverse out-of-domain test datasets with domain shifts and missing contrasts, including the public MICCAI 2016 and UMCL datasets, as well as a private multisite dataset. These test datasets exhibit domain shifts and/or missing contrasts caused by variations in acquisition protocols, scanner types, and imaging artifacts arising from imperfect acquisition. Our code is available at https://github.com/Jinwei1209/UNISELF.

## 1. Introduction

Multiple sclerosis (MS) is characterized by chronic inflammatory demyelination and neurodegeneration of the central nervous system (Haider et al., 2016). Magnetic resonance imaging (MRI) is commonly used for diagnosing and monitoring MS in clinics due to its sensitivity to focal tissue inflammation (Filippi et al., 2019). In MS, demyelinating white matter lesions typically appear hyperintense on T2-weighted (T2w) and T2-weighted Fluid-Attenuated Inversion Recovery (FLAIR) images, and hypointense on T1-weighted (T1w) images. Advancements in MRI, such as higher spatial resolution, improved signal-to-noise ratio, and biomarkers specific to the pathology of MS disease (e.g. iron rim lesions Dal-Bianco et al., 2017) make analyzing the longitudinal evolution of the progression of the lesion more informative. These advancements improve the reliability of treatment evaluations (Kolb et al., 2022). Accurate lesion mask delineations serve as a foundational step for quantifying lesion volumes and extracting advanced biomarkers relevant to lesion progression, such as iron rim lesions, which are linked to chronic active inflammation in MS (Kaunzner et al., 2019). However, manual delineation of MS lesions faces challenges in efficiency and reproducibility due to their heterogeneous shape, size, and location (as shown in Fig. 3). In addition, inter-rater variability (Carass et al., 2017b, 2020; Commowick et al., 2021b; Styner et al., 2008; Zijdenbos et al., 1994) and lack of consistency across imaging sites and protocols (Carass et al., 2024) make manual segmentation particularly difficult, especially in multi-center and retrospective studies. To address these challenges, researchers have focused on developing automated lesion segmentation methods for many years. However, variations in image quality across scanners (van Nederpelt et al., 2024) and the limited availability of manually delineated datasets for training learning-based methods (Carass et al., 2017a) pose challenges for automated segmentation methods.

Automated lesion segmentation methods can be categorized as classical or learning-based depending on whether image features are hand-crafted or learned from data. For classical methods, early approaches included the use of hand-crafted image features such as atlas-based topology priors with an additional lesion class (Shiee et al., 2010) and probabilistic models for lesion classification and growth (Schmidt et al., 2012), as well as more recent probabilistic segmentation models like SAMSEG (Cerri et al., 2021). For learning-based methods, early feature extraction techniques have been used, such as sparse coding of image patches through dictionary learning (Weiss et al., 2013). With the emergence of deep learning, recent research has primarily involved training convolutional neural network models using manually delineated labels to learn image features. Pioneering work includes 3D patch-based cascaded architecture (Valverde et al., 2017), 2D slice-based multi-branch architecture (Aslani et al., 2019), and 3D volume-based encoder-decoder architecture (Brosch et al., 2016). Since then, deep learning methods have demonstrated improved performance over earlier methods in terms of both accuracy and efficiency (Jiang et al., 2023; Ma et al., 2022), with state-of-the-art models such as LST-AI (Wiltgen et al., 2024) and HD-MS-Lesions (Brugnara et al., 2020) being publicly released along with their trained weights.

Some recent work has focused on improving segmentation accuracy when trained with limited data with manual delineations by adapting the widely used U-Net (Ronneberger et al., 2015) architecture. U-Net has the advantages of both multiscale feature representation and skip connections, enabling accurate MS lesion segmentation. For example, the best-performing published lesion segmentation methods evaluated using the 2015 ISBI longitudinal MS lesion segmentation challenge data (Carass et al., 2017b,a) are Tiramisu (Zhang et al., 2019a) and ALL-Net (Zhang et al., 2021a). In Tiramisu, the convolutional layer in the U-Net was replaced with a densely connected convolutional layer (Jégou et al., 2017) to improve feature learning. In ALL-Net, a coordinate convolutional layer was incorporated into their U-Net to capture anatomical information. Other modifications in network architecture include the addition of attention mechanisms in U-Net to improve segmentation accuracy, such as slice-wise (Zhang et al., 2019b) and folded attention U-Net for 3D volumes (Zhang et al., 2021b), U-Net with squeeze and attention modules (Rondinella et al., 2023), and attention-gated U-Net (Hashemi et al., 2022). Those variants of U-Net have been reported to improve segmentation accuracy for in-domain tests, such as the ISBI challenge, when the data distributions between training and test are identical. In parallel, the nnU-Net framework (Isensee et al., 2021) emerged as a self-configuring U-Net-based architecture that has become a widely adopted state-of-the-art baseline across various medical image segmentation tasks, including MS lesion segmentation. For example, nnU-Net has demonstrated strong performance in more recent MS lesion segmentation challenges, such as in the MS new lesion segmentation challenge MSSEG-2 (Commowick et al., 2021a).

In addition to improving accuracy on in-domain tests when trained with limited data, it is equally important to deal with out-of-domain shifts when the test data distribution deviates from the training. Domain shifts in MRI for MS lesions include variations in image contrast due to scanner or protocol differences, as well as imaging artifacts introduced during acquisition. Those domain shifts can potentially cause generalization errors (Quiñonero-Candela et al., 2022) and reduce the accuracy of trained segmentation models. There are three philosophies for addressing domain shifts for MS lesion segmentation: domain harmonization, adaptation, and generalization. For domain harmonization, the variation in contrast between scanners is mitigated by using synthesis-based harmonization techniques (Roy et al., 2010; Dewey et al., 2019; Zuo et al., 2022, 2023; Gebre et al., 2023). Harmonized images are expected to maintain consistent contrast in both training and test data thus prevent domain shifts; Carass et al. (2024) demonstrated that this is at least true with respect to manually generated delineations. For domain adaptation, pre-trained models are adapted to a target test domain using new labeled data from that domain. Example adaptation methods include one-shot adaptation (Valverde et al., 2019) and harmonization-enriched domain adaptation (Zhang et al., 2024a). Additionally, unsupervised domain adaptation can be performed without requiring target domain labels (Gérin et al., 2024). For domain generalization, invariant/adaptive features are imposed to reduce domain shifts. Methods such as spatially adaptive sub-networks (Kamraoui et al., 2022), domain-invariant latent features (Zhao et al., 2021; Aslani et al., 2020), contrast-adaptive modeling (Cerri et al., 2021), domain generalization augmentation (Zhang et al., 2023a), and federated learning (Liu et al., 2023) have been proposed, contributing to more reliable automated MS lesion segmentation in clinical and multisite settings. Recent efforts have further emphasized the importance of addressing real-world domain shifts specific to MS lesion segmentation through approaches such as test-time training (Gérin et al., 2024), domain randomization (Billot et al., 2023; Laso et al., 2024), and benchmarking on distribution shifts (Malinin et al., 2022), with methods including deep ensembles (Lakshminarayanan et al., 2017).

The handling of varying sets of available contrast-weighted MR images across different datasets is a crucial generalization ability for MS lesion segmentation in clinical practice. For example, the 2015 ISBI challenge (Carass et al., 2017b) dataset provides T1w, T2w, proton-density-weighted (PDw), and FLAIR images with every subject, but it is not common for all four of these contrasts to be available in clinical practice. T2w or FLAIR images are essential for identifying hyperintense demyelinating lesions, whereas the T1w image mainly provides complementary information for tissue characterization (Wattjes et al., 2015; Filippi et al., 2016). Therefore, missing both T2w and FLAIR may lead to lower segmentation reliability, while missing T1w is less critical for lesion segmentation. Most prior MS segmentation methods assume that the same set of contrasts is available during both training and testing and fix the network input accordingly, which limits their flexibility for a broader clinical deployment. Only a few studies have explored the ability of networks to handle missing contrasts. Feng et al. (2019) applied a contrast dropout training strategy by randomly replacing a subset of all available contrasts with constant values (e.g., all zeros) when forming the training inputs without changing the network architecture. ModDrop+ + (Liu et al., 2022) improved contrast dropout performance using a dynamic network architecture and a co-training loss involving full and missing contrasts during training. In Zhang et al. (2023a), contrast dropout was combined with domain generalization augmentation to further improve generalization performance and combat missing contrasts. Recently, WMH-SynthSeg (Laso et al., 2024) has been introduced as a domain-randomized CNN capable of segmenting white matter hyperintensities and brain anatomy from scans of any contrast and resolution, including low-field MRI, without retraining.

Despite the success of deep learning methods for MS lesion segmentation, few have been shown to simultaneously achieve strong in-domain accuracy, robust out-of-domain generalization, and the ability to handle missing contrasts. Furthermore, their performance has not been satisfactory, especially with limited single-source training data. For example, Tiramisu (Zhang et al., 2019a) and ALL-Net (Zhang et al., 2021a) achieved the highest accuracy in the ISBI challenge, but used fixed multicontrast inputs and did not validate multisite generalization. DeepLesionBrain (Kamraoui et al., 2022) demonstrated good generalization to unseen domains, but it had lower in-domain accuracy than Tiramisu and used fixed network inputs. Data augmentation with contrast dropout (Zhang et al., 2023a) also exhibited good generalization, but its in-domain accuracy was not validated. ModDrop+ + (Liu et al., 2022) trained a unified model capable of handling various input contrasts while maintaining accuracy compared to independently trained models, but its generalization to unseen domains was unknown. Image harmonization methods such as HACA3 (Zuo et al., 2023) have the potential to improve generalization in downstream lesion segmentation by harmonizing multicontrast images and imputing missing contrasts. However, the generalization of HACA3 itself should be guaranteed before its application, and training a generalizable HACA3 model requires a multisite training dataset, which may not be readily available to all institutions.

To address the aforementioned gap, we propose UNISELF (**U**nified **N**etwork with **I**nstance normalization and **S**elf-**E**nsembled **L**esion **F**usion), a method that achieves state-of-the-art performance for MS lesion segmentation both in-domain and out-of-domain under the constraints of training on a limited sized single source dataset with missing contrasts at inference. To train the model, we used the publicly available single-site 2015 ISBI challenge training dataset (Carass et al., 2017b), which contains only 5 subjects with an average of 4.2 longitudinal scans per subject in training. Our results demonstrate superior in-domain accuracy on the ISBI challenge test dataset, as well as strong generalization to domain shifts and missing contrasts across multiple multisite test datasets. This work extends our conference paper (Zhang et al., 2024b) and includes the following contributions:

1. UNISELF includes a novel self-ensembled lesion fusion strategy that augments multi-orientation MRI inputs and ensembles the augmented outputs using a two-step lesion detection and growth approach to improve accuracy;
2. UNISELF leverages test-time instance normalization (TTIN) that normalizes latent features for each test input to improve model generalization in handling domain shifts and varying MRI contrasts;
3. Trained solely on the limited single-source ISBI dataset, UNISELF achieves state-of-the-art segmentation performance both in-domain and out-of-domain, demonstrating superior generalization across heterogeneous multisite test data compared with benchmark methods.

Our code is available at https://github.com/Jinwei1209/UNISELF.

## 2. Related works

### 2.1. Ensemble learning

Ensemble learning is a technique that combines multiple diverse learning algorithms or models to capture a wider range of unique patterns and features compared to individual algorithms (Opitz and Maclin, 1999; Kuncheva and Whitaker, 2003), thus enhancing robustness and accuracy. In MS lesion segmentation, prior works such as Tohidi et al. (2022) trained multiple models with diverse architectures to achieve better results compared to individual models. However, the performance of the ensemble in Tohidi et al. (2022) was not compared to benchmark methods and datasets like Tiramisu (Zhang et al., 2019a) and ALL-Net (Zhang et al., 2021a) in the 2015 ISBI challenge. Furthermore, selecting architectures to improve model diversity can be a laborious and arbitrary process, sometimes even resulting in worse performance compared to dedicatedly designed single models (Zhang et al., 2021a).

### 2.2. Test-time augmentation

Data augmentation addresses the overfitting of trained models by enhancing the size and variety of limited training data by applying multiple transformations such as flips and rotations to each sample during training (Shorten and Khoshgoftaar, 2019). Test-time augmentation (TTA) applies multiple transformations to test data in the same way as during training, thus generating a more robust prediction from multiple predictions compared to a single prediction without augmentation (Shorten and Khoshgoftaar, 2019). TTA resembles ensemble learning in the data space and has been used in medical image segmentation (Moshkov et al., 2020; Wang et al., 2019; Henschel et al., 2020; Isensee et al., 2021). For MS lesion segmentation, a widely used TTA approach generates three 3D lesion masks, each derived from one of the three cardinal planes (axial, sagittal, and coronal), followed by voxel-wise majority voting to obtain the final 3D mask (Zhang et al., 2019a). However, this aggregation strategy does not consider the multi-instance nature of MS lesion detection and segmentation. Moreover, comprehensive multi-orientation augmentations including flipping and rotation are not considered in this approach.

### 2.3. Latent feature regulation

Batch normalization (BN) (Ioffe and Szegedy, 2015) has been widely used in MS lesion segmentation models under the assumption that there are no domain shifts between training and test data (Zhang et al., 2019a, 2021a). However, BN stores domain-specific training feature statistics to normalize test-time features, which may not generalize well to out-of-domain test datasets. To improve robustness under domain shift, Nado et al. (2020) demonstrated that recomputing BN statistics at prediction time for each test batch can effectively improve model robustness. In the context of MS lesion segmentation, latent feature regulation is particularly important because feature distributions can vary substantially across sites, scanners, and contrast settings. Prior works in generalizable MS lesion segmentation have attempted to mitigate this issue by learning domain-invariant features across sites (Zhao et al., 2021; Aslani et al., 2020), but these approaches typically require multisite training data with lesion mask labels, which may be difficult to obtain in practice.

### 2.4. Handling missing contrast

Handling missing MRI contrasts is critical for deploying segmentation models in real-world clinical environments, where not all contrast-weighted MR images are consistently available. Early work by Feng et al. (2019) proposed contrast dropout, which trains the network with randomly dropping input contrasts (replacing them with zeros) to improve robustness to missing contrasts at inference. This strategy was extended in ModDrop+ + (Liu et al., 2022), which introduced a dynamic network architecture and a co-training loss between full and partial contrast inputs to learn contrast-invariant features. Zhang et al. (2023a) further enhanced generalization by combining contrast dropout with domain generalization augmentation. More recently, WMH-SynthSeg (Laso et al., 2024) adopted a domain-randomization strategy to enable segmentation of white matter hyperintensities and brain anatomy from scans of any contrast and resolution, including low-field MRI, without retraining. However, these approaches either do not explicitly address the intrinsic distribution shifts caused by varying input contrasts or require complex training procedures.

## 3. Method

We propose UNISELF to address the limitations of existing test-time augmentation (data ensemble learning), latent feature regulation, and missing contrast handling methods in improving MS lesion segmentation accuracy and generalization. Trained on limited single-site data, UNISELF simultaneously achieves high in-domain accuracy, strong out-of-domain generalization, and robustness to contrast variability. Section 3.1 describes the training stage of UNISELF, which involves training a 2.5D U-Net backbone with spatial augmentation and contrast dropout (CD) to expose the network to diverse spatial orientations and all possible missing-contrast scenarios. Sections 3.2 and 3.3 then present the two key building blocks of UNISELF in detail: (1) self-ensembled lesion fusion and (2) test-time instance normalization, respectively.

### 3.1. Training stage

This section describes the training setup of UNISELF, including multi-orientation augmentation for 2.5D processing and the strategy for handling missing contrasts using CD. A single U-Net (Ronneberger et al., 2015) is trained to process multicontrast 3D MRI data in a slice-wise 2.5D manner with extensive spatial and input contrast augmentations. The goal of this training stage is to expose the network to diverse spatial orientations and all possible contrast-missing scenarios, serving as a preparatory step for the proposed self-ensembled lesion fusion and test-time instance normalization described in Sections 3.2 and 3.3.

#### Multi-orientation augmentation in training

**Fig. 1.**
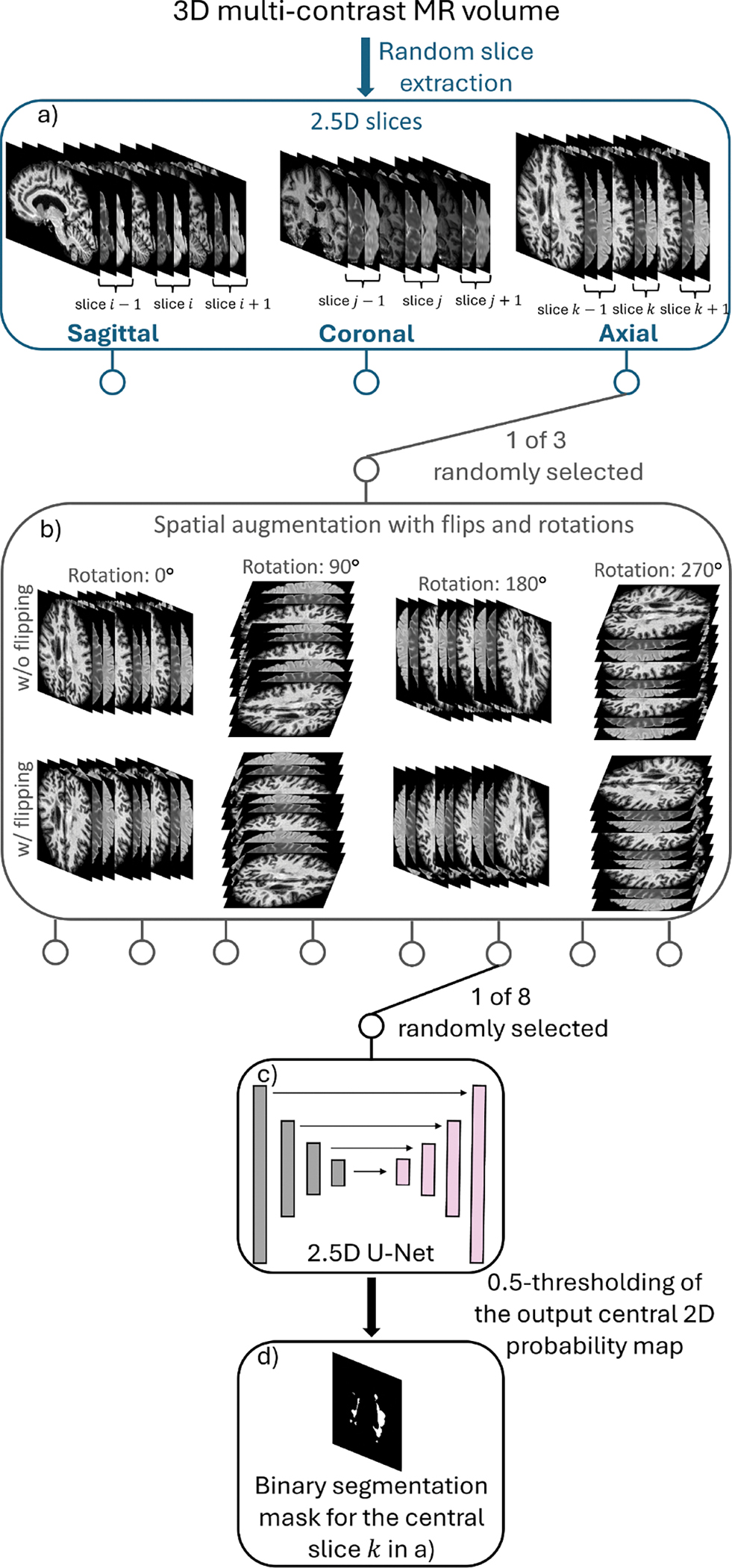
An illustration of the spatial augmentation, network input, and network output during training in UNISELF.

In UNISELF, a single U-Net is trained to slice-wise process multi-contrast 3D MRI volumes with multi-orientation augmentation. Fig. 1 presents an overview of the multi-orientation data augmentation process applied during training. In Fig. 1a, three adjacent 2D slices of multicontrast MRI, concatenated along the channel dimension with zero-padding at the boundaries (referred to as 2.5D input Zhang et al., 2019a), are extracted from a random selection of the three cardinal planes: axial, sagittal, or coronal. In Fig. 1b, a randomly selected augmentation from 8 possible augmentations, including flips (vertical and horizontal) and rotations (0°, 90°, 180°, and 270°), is applied to the extracted 2.5D input. These augmentations alter the spatial configuration of the 2.5D input without changing its intensity values, preserving the original image information while enhancing the diversity of inputs encountered by the network during training. In Fig. 1c, the augmented 2.5D input is fed into the network to generate the probabilistic output map of the central 2D slice. In Fig. 1d, a binary segmentation mask is obtained from the augmented 2.5D input by applying a threshold of 0.5 to the output probabilistic map, classifying pixels with values above 0.5 as lesion and those below as non-lesion areas. The ground truth lesion mask of the central slice undergoes the same augmentation process as in Fig. 1a and b and is used in the loss function for backpropagation.

#### Handling missing contrasts with CD

Contrast dropout (CD) (Feng et al., 2019) is applied to expose the network to all possible missing-contrast scenarios during training. In each iteration, a random subset of available contrasts (T1w, T2w, PDw, or FLAIR) is replaced with all-zero images, simulating situations where one or more contrasts may be unavailable. This strategy exposes the network to all possible contrast combinations during training, serving as a preparatory step for handling missing contrasts during inference.

### 3.2. Self-ensembled lesion fusion

In UNISELF, we introduce a novel self-ensembled lesion fusion strategy to improve model accuracy. Unlike existing TTA approaches in medical image segmentation such as those used in *FastSurfer* (Henschel et al., 2020) and *nnU-Net* (Isensee et al., 2021), which aggregate multi-orientation TTA predictions via (weighted) averaging, we propose a novel two-step lesion detection and connected growth approach to fuse multi-orientation TTA predictions. Specifically, the first step, “lesion detection”, identifies lesioned voxels with high confidence. The second step, “connected lesion growth”, expands these detected lesions to neighboring voxels to recover the full lesion extent. Together, these two steps form our “lesion fusion” strategy, which aggregates multi-orientation TTA predictions into a final segmentation using a single (unified) model. This lesion fusion strategy forms the first major methodological contribution of our work, as detailed in the following sections.

#### Confidence map at test-time

At test-time, a *confidence map* is generated through comprehensive multi-orientation processing and aggregation of the same multicontrast 3D volume using the trained segmentation model. Specifically, the eight spatial augmentations shown in Fig. 1b are applied to each of the three cardinal plane 3D volumes, resulting in $N_{M}=8\times 3=24$ augmented multicontrast 3D volumes. For any augmented input volume with index $i\;\left(1\leq i\leq N_{M}\right)$, the trained network processes the volume in a 2.5D fashion, where 2D binary segmentation masks are generated slice-by-slice and subsequently stacked to form a 3D binary mask corresponding to the augmented input volume. The resulting 3D binary mask is then flipped and rotated back into the original space, denoted as $M_{i}(r)$, where r represents the spatial coordinate of a 3D voxel in the original space. A confidence map C(r) with integer values between 0 and $N_{M}$ is then generated by adding $N_{M}$ 3D binary masks in the original space:

(1) $$C\left(r\right)=\sum_{i=1}^{N_{M}}M_{i}\left(r\right),\forall r.$$

**Fig. 2.**
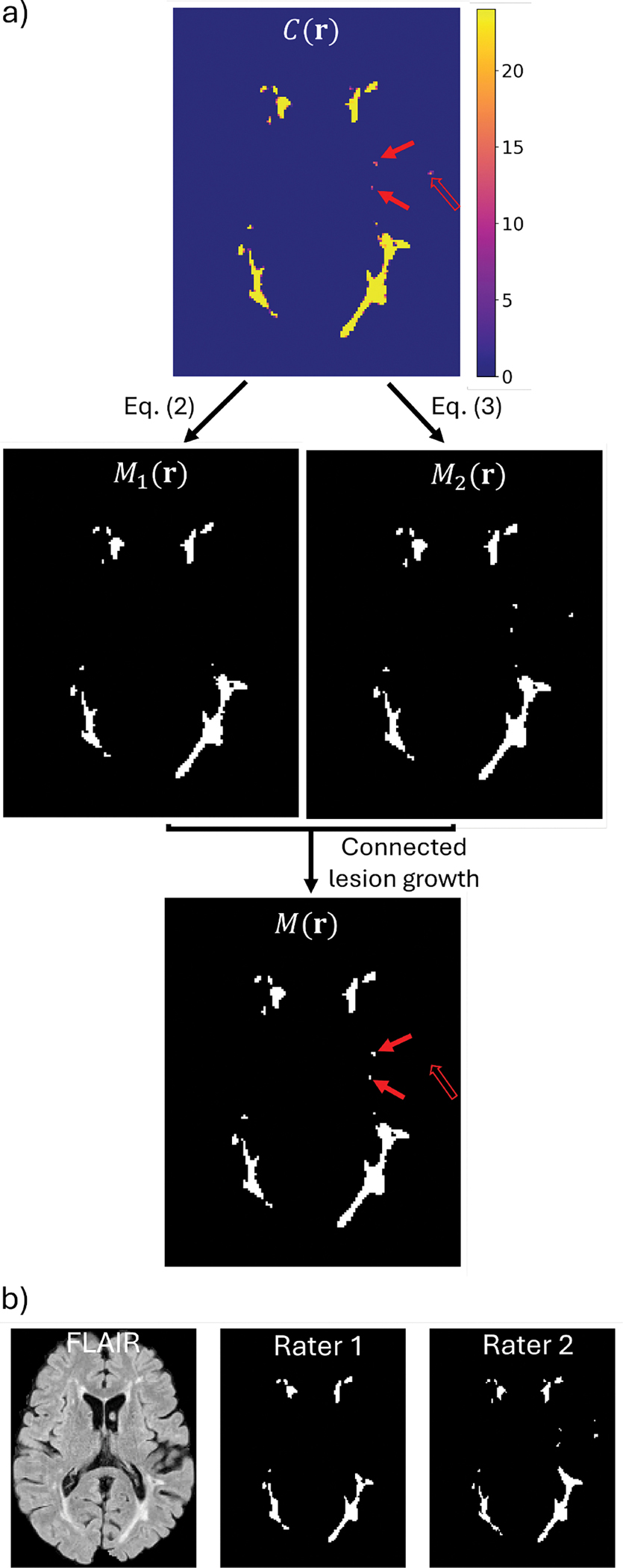
Illustration of the proposed self-ensembled lesion fusion strategy in UNISELF. (a): Confidence map C(r) (top), two binary lesion masks $M_{1}(r)$ and $M_{2}(r)$ (middle), and final binary lesion mask M(r) (bottom). (b): Corresponding FLAIR image with lesion delineations from two raters as reference.

This confidence map indicates the number of times that the network, trained with all cardinal planes, rotations, and flips, predicts a voxel as part of a lesion. Fig. 2a shows an example $C\left(r\right)$ as a heatmap ranging between integer values of 0 and 24. For example, $C\left(r\right)=24$ means that the model consistently predicts the voxel at r as belonging to a lesion under all spatial augmentations. We use $C\left(r\right)$ to derive the final lesion segmentation mask through a two-step process of lesion detection and connected lesion growth, as detailed in the next section.

#### Lesion detection and connected growth

After obtaining C(r) using Eq. (1), the next step applies two thresholds $\tau_{1}$ and $\tau_{2}$, where $\tau_{1}\geq \tau_{2}$, on C(r) to generate two binary segmentation masks:

(2) $$M_{1}(r)=\left\{\begin{array}{ll} 1 & C(r)>\tau_{1},\\ 0 & \text{otherwise} \end{array}\;\forall r,\right.$$

and

(3) $$M_{2}(r)=\left\{\begin{array}{ll} 1 & C(r)>\tau_{2},\\ 0 & \text{otherwise} \end{array}\;\forall r.\right.$$

Fig. 2a shows an example of $M_{1}(r)$ and $M_{2}(r)$. Because $\tau_{1}\geq \tau_{2}$, the binary region defined by $M_{2}(r)$ always includes that defined by $M_{1}(r)$. Moreover, since the voxels in $M_{1}(r)$ have greater certainty of belonging to a lesion, they are considered to belong to lesions without further evaluation and are referred to as *detected lesions*.

We use $M_{2}(r)$ in a *connected lesion growth* step to provide candidates to also include within the detected lesions. The connected lesion growth step produces the final segmentation M(r) by iteratively expanding the detected lesions defined by $M_{1}(r)$ to include additional spatially connected voxels identified in $M_{2}(r)$. We use 26-connectivity for this growth process. Fig. 2a shows an example of the final segmentation M(r) after connected lesion growth, where segmentation masks of small lesions (denoted by solid red arrows, present in other axial slices in $M_{1}(r)$ that are not shown in Fig. 2a) are grown and refined in M(r), while lesions not detected in $M_{1}(r)$ (denoted by hollow red arrows) do not appear in M(r) either. Fig. 2b shows the corresponding FLAIR image with lesion masks delineated by two raters as reference. We experimentally determine optimal values for $\tau_{1}$ and $\tau_{2}$ (see Section 6.3) to ensure their effectiveness in the proposed self-ensemble strategy.

### 3.3. Test-time instance normalization (TTIN)

To improve UNISELF generalization, we propose to leverage TTIN to address potential latent feature mismatches caused by (1) out-of-domain shifts and (2) varying input contrast: (1) Specifically, we demonstrate that TTIN mitigates feature statistics shifts caused by out-of-domain shifts (As shown in Fig. 8a); (2) Most importantly, we observe that varying combinations of input contrasts also induces shifts in feature statistics, even when such combinations were present via contrast dropout during training (also shown in Fig. 8a). To mitigate this, we propose the use of TTIN to recalibrate instance normalization statistics at inference time on a per-input basis, preserving invariant feature representations across contrast variations. Integrating TTIN with contrast dropout forms the second major methodological contribution of our work, as explained in the following sections.

#### Batch normalization in training and testing

The distribution of latent features in any layer can be regulated by Batch normalization (BN) to stabilize and accelerate stochastic gradient descent optimization (Ioffe and Szegedy, 2015). During mini-batch training, for a layer with feature map $x=\left(x_{1},x_{2},\cdots ,x_{c}\right)\in R^{n\times c}$, where n denotes the batch size, and c denotes the feature/channel dimension, BN transforms x in a feature-wise manner with the following steps:

(4a) $${\overset{ˆ}{x}}_{j}=\frac{x_{j}\;-\;E\left[x_{j}\right]}{\sqrt{Var\left[x_{j}\right]\;+\;\epsilon }},$$

(4b) $$y_{j}=\gamma_{j}{\overset{ˆ}{x}}_{j}+\beta_{j}$$

where $\gamma_{j}$ and $\beta_{j}$ in Eq. (4b) are learnable parameters for each feature/channel j∈{1,⋯,c}. During BN training, the moving averages of $E\left[x_{j}\right]$ and $Var\left[x_{j}\right]$ over all mini-batches are stored and denoted as $E_{MA}\left[x_{j}\right]$ and ${Var}_{MA}\left[x_{j}\right]$, respectively. At test-time, $E\left[x_{j}\right]$ and $Var\left[x_{j}\right]$ in Eq. (4a) are replaced with $E_{MA}\left[x_{j}\right]$ and ${Var}_{MA}\left[x_{j}\right]$ for all test data, and $\gamma_{j}$ and $\beta_{j}$ in Eq. (4b) are retained from training (Ioffe and Szegedy, 2015).

#### Domain shifts in feature space

In test scenarios with domain shifts such as contrast variation or missing contrast in multisite MRI data, a mismatch in latent features between training and test datasets may occur. A visualization of such latent space feature mismatch normalized by $E_{MA}\left[x_{j}\right]$ and ${Var}_{MA}\left[x_{j}\right]$ is presented in Section 6.2 with a detailed experimental setup. Distinct clusters corresponding to each test site are observed in the latent space features of both shallow and deep layers in a U-Net. This indicates a mismatch across different test sites resulting from domain shifts in the test data distribution relative to the training data distribution (i.e., $E_{MA}\left[x_{j}\right]$ and ${Var}_{MA}\left[x_{j}\right]$).

#### TTIN to handle domain shifts

Given the potential mismatch between training and test domain features normalized with $E_{MA}\left[x_{j}\right]$ and ${Var}_{MA}\left[x_{j}\right]$, we propose to use *instance-specific* statistics, i.e., test-time instance normalization (TTIN), for any given input at test-time to handle domain shifts. Specifically, $E\left[x_{j}\right]$ and $Var\left[x_{j}\right]$ in Eq. (4a) are computed for each test input (per 2.5D slice, using a batch size of one during inference), instead of using $E_{MA}\left[x_{j}\right]$ and ${Var}_{MA}\left[x_{j}\right]$ from training. The parameters $\gamma_{j}$ and $\beta_{j}$ in Eq. (4b) are retained from the training phase. In Section 6.2 we demonstrate that the features normalized by TTIN appear mixed without distinct clusters, indicating improved alignment across various test sites.

Since $\gamma_{j}$ and $\beta_{j}$ are retained from training, feature normalization methods on the training of $\gamma_{j}$ and $\beta_{j}$ in Eq. (4b) may affect the performance of TTIN. Different feature normalization methods for training $\gamma_{j}$ and $\beta_{j}$ in Eq. (4b) and their impact on TTIN is assessed in the experiments section (see Sections 6.4 and 6.5), including BN, IN, and conditional IN (CondIN) (Dumoulin et al., 2016), where CondIN involves conditional $\gamma_{j}$ and $\beta_{j}$ for each input contrast combination. In UNISELF, CondIN is adopted, based on cross-validation results comparing the performance of TTIN trained with BN, IN, and CondIN (see Section 4.3).

### 3.4. Implementation details

We use U-Net (Ronneberger et al., 2015) as the backbone architecture for UNISELF. Specifically, we adopt a 5-level encoder-decoder U-Net with 64, 128, 256, 512, and 1024 feature channels at each level, respectively. Each level contains two sequential blocks of 3×3 convolution, ReLU activation, and feature normalization. Downsampling is performed using 2×2 max pooling, while upsampling is performed using nearest-neighbor interpolation followed by a convolution. Training details are listed below:

- Prior to the multi-orientation augmentation in Fig. 1, 3D elastic or affine transformations were randomly applied using TorchIO (Pérez-García et al., 2021) (with a probability of 75% for each multicontrast 3D volume) to augment brain tissue and lesion shapes.
- For each training batch, individual samples were drawn by first randomly selecting a subject and then randomly extracting a 2.5 D slice that contained at least one lesion voxel in the central slice. All samples in the batch were taken from the same imaging plane (e.g., axial) and underwent identical spatial augmentations, including random rotation and flipping.
- The $L_{2}$ loss was used between the predicted probabilistic segmentation map and the label for back-propagation, as it demonstrated better overall performance compared to the Dice and focal losses in 2.5D setting (Zhang et al., 2019a).
- Random sampling of two-rater labels was employed (Jungo et al., 2018).
- Contrast dropout (CD) (Feng et al., 2019) was applied by randomly replacing a subset of available contrasts in each training batch with all-zero images.
- The Adam optimizer (Kingma and Ba, 2014) was used to update the network weights with an initial learning rate of 10^−4^, a mini-batch size of 12, and 150 epochs with 300 iterations per epoch. The model from the last training epoch was selected as the final model for evaluation.

## 4. Experimental setup

### 4.1. Datasets

#### 4.1.1. Training dataset: Public 2015 ISBI challenge

The 2015 ISBI longitudinal MS segmentation challenge (Carass et al., 2017b) training dataset was used to train all models in this work. This training dataset consists of 21 time points from 5 subjects acquired using a single scanner. Four contrasts (T1w, T2w, PDw, and FLAIR) with two expert delineations are available for all time points. All data were rigidly registered to the 1mm^3^ MNI space using MIPAV’s optimized automatic registration^1^ followed by brain extraction and dura removal,^2^ as well as N4 bias field correction.^3^

#### 4.1.2. Test dataset I: Public 2015 ISBI challenge

The 2015 ISBI challenge test dataset consists of 61 time points from 14 subjects acquired using the same scanner as the training dataset, with the same four contrasts and pre-processing steps (Carass et al., 2017b). Expert delineations are not available to the public. Instead, the test performance is assessed by submitting the segmentation results to the challenge website to obtain a score.

#### 4.1.3. Test dataset II: Public 2016 MICCAI challenge

The 2016 MICCAI MS segmentation challenge (Commowick et al., 2018) contains a training dataset of 15 subjects acquired from three different scanners. Five contrasts (T1w, T1w with gadolinium, T2w, PDw, and FLAIR) with a consensus of seven expert delineations are available for all subjects. For each subject, each contrast was denoised with the non-local means algorithm (Coupé et al., 2008), followed by rigid body registration to the corresponding FLAIR contrast, skull stripping, and N4 bias field correction. Rigid registration to the ICBM 152 MNI space was applied to each contrast. T1w images with gadolinium contrast were not used to ensure that the input multicontrast images at test-time are a subset of those used in training.

#### 4.1.4. Test dataset III: Public UMCL data

The UMCL (University Medical Center Ljubljana) dataset (Lesjak et al., 2018) contains 30 subjects acquired using a single scanner. Four contrasts (T1w, T1w with gadolinium, T2w, and FLAIR) with a consensus of three expert delineations are available for all subjects. All data were registered to the 1mm^3^ MNI space with N4 correction. T1w images with gadolinium contrast were not used.

#### 4.1.5. Test dataset IV: Private multisite data

In our study, a private multisite data set was used for additional generalization validation. This multisite dataset contains 93 subjects acquired from 9 scanners (sites) in a clinical setting, with various available T1w, T2w, PDw, and FLAIR contrasts, but does not include manual delineations of lesions. All subjects had a T1w scan, along with at least one additional contrast (T2w, PDw, or FLAIR). No subject had a T1w-only acquisition. Among the 93 subjects, the acquisition resolution statistics ([frequency, phase, slice encodings]) for each MRI contrast are summarized as follows:

- **T1w**: 92 3D scans ([1.02 ± 0.08, 1.02 ± 0.08, 1.04 ± 0.08] mm^3^), 1 2D scan ([1.07, 0.75, 5.00] mm^3^), and 0 missing.
- **T2w**: 16 3D scans ([0.98 ± 0.03, 0.98 ± 0.03, 1.00 ± 0.00] mm^3^), 72 2D scans ([0.86 ± 0.19, 0.80 ± 0.23, 3.22 ± 0.85] mm^3^), and 5 missing.
- **PDw**: 0 3D scans, 63 2D scans ([0.89 ± 0.16, 0.85 ± 0.23, 2.87 ± 0.51] mm^3^), and 30 missing.
- **FLAIR**: 78 3D scans ([1.01 ± 0.06, 0.99 ± 0.03, 1.02 ± 0.08] mm^3^), 13 2D scans ([0.88 ± 0.11, 0.88 ± 0.06, 3.38 ± 1.07] mm^3^), and 2 missing.

**Fig. 3.**
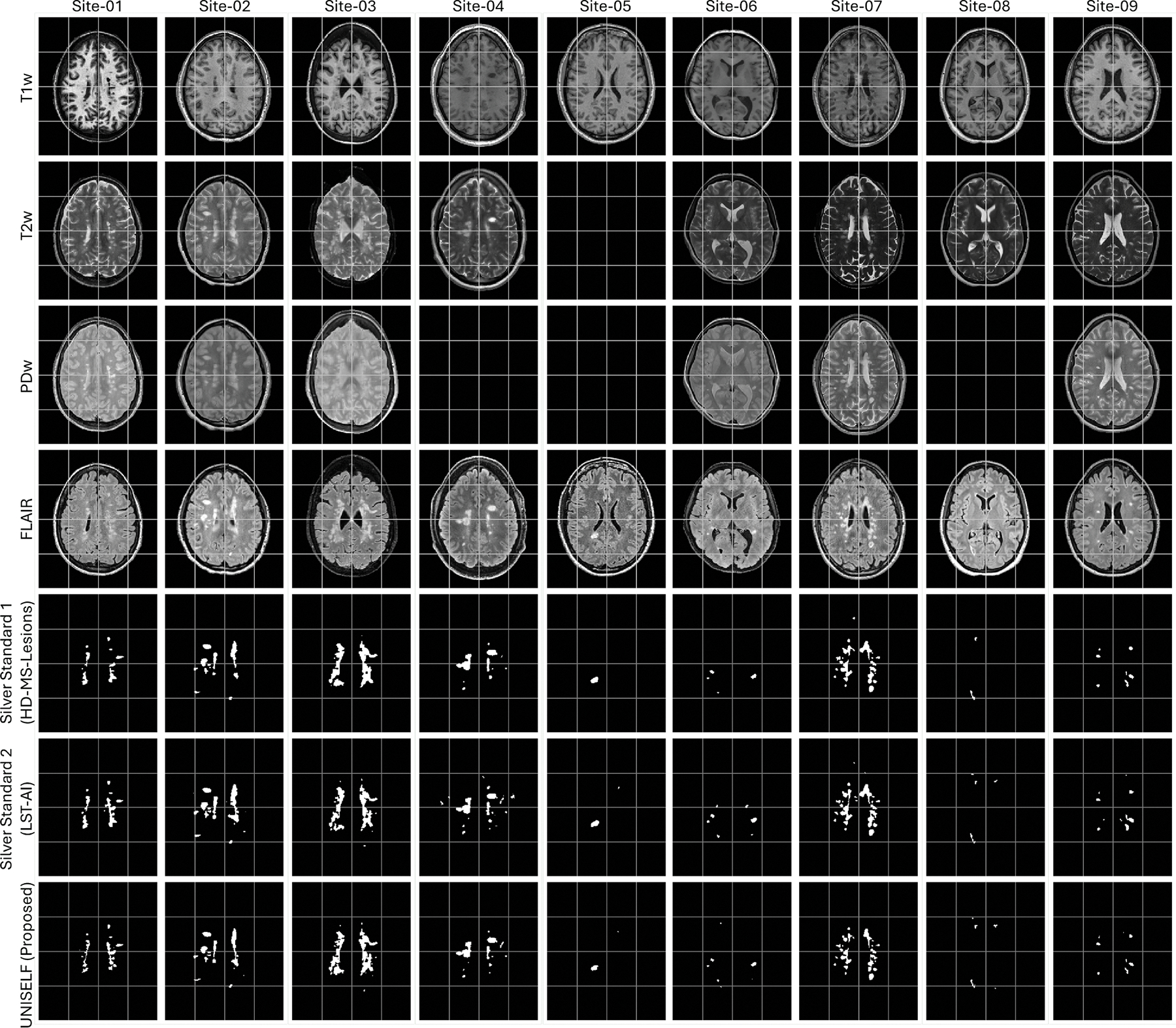
Example images from the private multisite test dataset with silver standard lesion segmentation labels and UNISELF predictions.

All data went through similar pre-processing steps as the ISBI data, with an additional super-resolution step using SMORE (Remedios et al., 2023; Zhao et al., 2020) on the multislice 2D acquired images before registration to the 1mm^3^ MNI space. Representative multicontrast images after pre-processing are shown in rows 1–4 of Fig. 3.

**Fig. 4.**
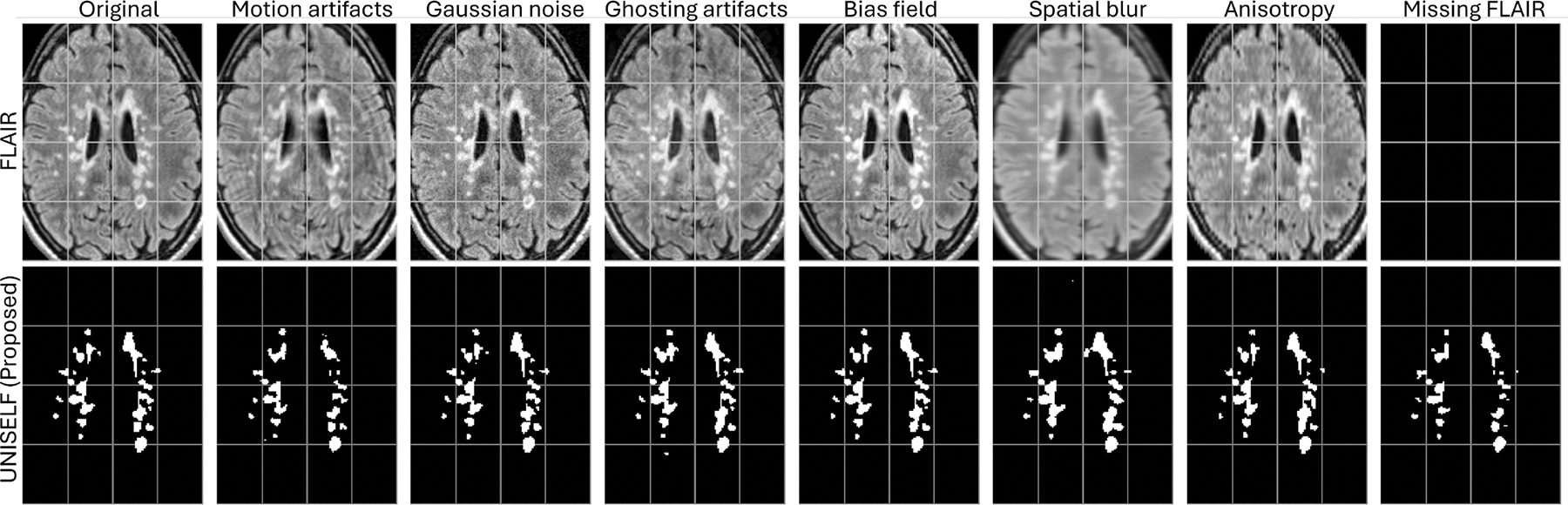
Example FLAIR images of ‘Site-07’ in Fig. 3 with various simulated artifacts and the corresponding UNISELF predictions.

To further validate the generalization performance of trained models to imaging artifacts, FLAIR images in the Test Dataset IV were corrupted with various artifacts, including motion, Gaussian noise, ghosting, bias field, spatial blur, and anisotropy, using the TorchIO data augmentation library (Pérez-García et al., 2021), as well as missing FLAIR. Examples of corrupted images of one representative subject are shown in Fig. 4.

#### 4.1.6. Silver standard delineation generation on test dataset IV

Since manual lesion delineations are not available for the Test Dataset IV, we have designed the following protocol to generate silver standard delineations:

- First, we employed HACA3 (Zuo et al., 2023) to impute missing contrasts for each subject in Test Dataset IV. To ensure the generalization of contrast-to-contrast mapping, the HACA3 model was trained jointly on the ISBI training dataset and Test Dataset IV in an unsupervised manner without using any lesion labels, solely for missing contrast imputation and entirely independent of the lesion segmentation model training.
- Second, we adopted two independent, publicly available models, LST-AI (Wiltgen et al., 2024) and HD-MS-Lesions (Brugnara et al., 2020), to generate two silver standard delineations on Test Dataset IV (examples shown in rows 5 and 6 of Fig. 3) after missing contrast imputation. These models were trained on large, heterogeneous MS datasets in their respective studies and require fixed input combinations: “T1w+FLAIR” for LST-AI and “T1w+T2w+FLAIR” for HD-MS-Lesions.
- Finally, we evaluated the segmentation performance of both the benchmark methods and *UNISELF* by averaging the segmentation scores (as defined in Eq. (5)) across the two independent silver standard delineations, in accordance with the ISBI challenge protocol (Carass et al., 2017b), which relies on annotations from two human raters.

Note that the missing contrast imputation using HACA3 was performed only for generating silver-standard references. All segmentation models were trained solely on the ISBI training dataset and evaluated on the original acquired contrasts of Test Dataset IV.

### 4.2. Benchmarks and performance evaluation

We included benchmarks *Tiramisu* (Zhang et al., 2019a), *ModDrop*+ + (Liu et al., 2022), *CD* (Feng et al., 2019), *DG* using both BN and IN (Zhang et al., 2023a). All benchmarks and the proposed *UNISELF* were trained solely on the ISBI 2015 training dataset (Carass et al., 2017b) using the same training strategies described in Section 3.4. Benchmark implementations were based on their publicly available network architecture code, with method-specific custom adaptations applied where needed. For training, we used the same 3D affine and elastic transformation, training data sampling, *L*_2_ loss function, random label sampling, optimizer, and final-epoch model selection across methods, to ensure a controlled comparison focused solely on the impact of architectural differences of these benchmarks. During inference, we used a batch size of one for all methods, including those relying on BN statistics and those using different TTIN variants (trained with BN, IN, and CondIN). Customized adaptations for each benchmark include:

- CD training was applied to both *CD* (Feng et al., 2019) and *ModDrop*+ + (Liu et al., 2022) baselines.
- Independent models, each corresponding to a fixed subset of input contrasts, were trained for *Tiramisu* (Zhang et al., 2019a).
- A co-training loss involving full and missing contrasts as proposed in *ModDrop*+ + (Liu et al., 2022) was applied during the training of *ModDrop*+ +.
- Domain generalization (DG) augmentation was applied during the training of the *DG* baseline using BN and IN (Zhang et al., 2023a), which included contrast dropout, imaging artifacts (motion, Gaussian noise, ghosting, bias field, spatial blur, and anisotropy) using TorchIO (Pérez-García et al., 2021), and gamma transformation to simulate contrast variation. This forms a conservative comparison against the *DG* baseline, which was exposed to artifact corruptions during training.
- Random sampling of 3D patches (patch size: 112 × 112 × 112) during training and sliding-window inference (step size: 56 × 56 × 56) were implemented for the *CD* baseline (Feng et al., 2019), which employed a 3D U-Net architecture.

We also benchmarked against *nnU-Net* (Isensee et al., 2021), following the recommended training commands provided in the official *nnU-Net* repository to ensure a standardized setup and reproducibility. Since CD is not supported in *nnU-Net*, we trained independent *nnU-Net* models, each corresponding to a fixed subset of input contrasts. We further compared our method with *WMH-SynthSeg* (Laso et al., 2024), an automatic whole-brain parcellation tool that includes white matter lesion segmentation. Following the official *WMH-SynthSeg* installation and inference instructions with trained weights,^4^ we performed benchmarking using single-contrast inputs based on the priority order “FLAIR > T2w > PDw > T1w,” whenever available. This order reflects the imaging contrasts most relevant to lesion delineation in the ISBI challenge (Carass et al., 2017b), where FLAIR was used for expert annotation, and T2-weighted contrasts (T2w/PDw) provide complementary lesion visualization. Other potential benchmarks such as *ALL-Net* (Zhang et al., 2021a) and *DeepLesionBrain* (Kamraoui et al., 2022) were not included due to the unavailability of their source code. All benchmark models except *nnU-Net* (Isensee et al., 2021) and *WMH-SynthSeg* (Laso et al., 2024) were trained using the same *L*_2_ loss and evaluated at the final epoch to ensure consistent optimization and model selection criteria. The *nnU-Net* (Isensee et al., 2021) framework, by design, employs a combination of Dice and cross-entropy losses and cross-validation for checkpoint selection. No additional tuning or selective checkpointing was applied for any method, ensuring consistency and fairness across all models.

Evaluation measures are the same as those used in the ISBI 2015 challenge (Carass et al., 2017b): Dice Similarity Coefficient (DSC), Precision (PPV), Sensitivity (TPR), Lesion-wise True Positive Rate (LTPR), Lesion-wise False Positive Rate (LFPR), Pearson’s correlation coefficient of the lesion volumes (VC), and a score by a weighted average of the above:

(5) $$\text{Score}=\frac{\text{DSC}}{8}+\frac{\text{PPV}}{8}+\frac{\text{LTPR}}{4}+\frac{1\;-\;\text{LFPR}}{4}+\frac{\text{VC}}{4}.$$

The scores of the 2015 ISBI challenge test were obtained by submitting segmentation results and receiving normalized scores computed by the challenge website (Carass et al., 2017b). The scores of the other test datasets were computed using Eq. (5). We employed the Wilcoxon signed-rank test (Rey and Neuhäuser, 2011), a non-parametric test for paired samples, to assess the statistical significance of performance differences. To account for multiple comparisons, we applied the Benjamini-Hochberg procedure (Hochberg and Benjamini, 1990) to control the false discovery rate (FDR-BH) at a significance level of 0.05, which was applied for both in-domain and out-of-domain evaluations, and was conducted separately within each subplot of the figures (Figs. 6, 7, S4 and S5) and each column of the tables (Tables 1, 2, S1–S12, S14–S15).

### 4.3. Cross-validation to finalize UNISELF configuration

To determine the final *UNISELF* configuration prior to testing, including the parameters $\tau_{1}$ and $\tau_{2}$ used in self-ensembled lesion fusion (Section 3.2), and the choice of normalization technique (i.e., BN, IN, or CondIN) used to train $\gamma_{j}$ and $\beta_{j}$ in Eq. (4b) for different TTIN variants (Section 3.3), we performed five-fold cross-validation on the ISBI challenge training set, holding out one subject out of five in each fold for validation. The validation criterion was the segmentation score defined in Eq. (5). Figure S1 shows the cross-validation scores from a grid search of $\tau_{1}$ and $\tau_{2}$ for models trained with BN (Fig. S1a), IN (Fig. S1b), and CondIN (Fig. S1c). The best validation performance was achieved with $\tau_{1}=16$ and $\tau_{2}=7$ using CondIN training, and this configuration was fixed for all subsequent experiments across all test sets.

## 5. Validation

### 5.1. Comparison in the ISBI test

Table 1 shows a comparison of *UNISELF* with all in-house trained benchmark models (detailed in Section 4.2), as well as *ALL-Net* (Zhang et al., 2021a) and *Tiramisu* (Zhang et al., 2019a), whose results are reported in their respective papers as two of the top-performing methods in the ISBI challenge. The finalized *UNISELF* configuration is determined through cross-validation, as described in Section 4.3. *UNISELF* achieved a comparable Score to *ALL-Net* and *Tiramisu* reported in their paper (first column), with lower PPV and LTPR, but higher DSC, TPR, and (1-LFPR) (remaining columns). Compared to all in-house trained benchmarks, *UNISELF* achieved a significantly higher Score (first column) than all other methods, followed by *nnU-Net*, which ranked second. Table 2 shows the comparison under the missing FLAIR condition, where *UNISELF* again achieved a significantly higher Score (first column) than all benchmarks, followed by *nnU-Net*. Although *nnU-Net* performs better on some voxel-wise metrics (DSC and TPR), *UNISELF* shows a consistent advantage in lesion-wise metrics (comparable or higher LTPR and lower LFPR). Because the ISBI Score is dominated by lesion-wise performance (Eq. (5)), this advantage directly yields *UNISELF*’s significantly higher overall ISBI Scores despite mixed voxel-wise results. In terms of inference speed, *UNISELF* requires 2 minutes and 30 seconds with a GPU memory usage of 656 MB on an NVIDIA Quadro RTX 5000. As a comparison, *nnU-Net* requires approximately 1 minute and 40 seconds and memory usage of 1736 MB on the same GPU.

### 5.2. Comparison in out-of-domain tests

**Fig. 5.**
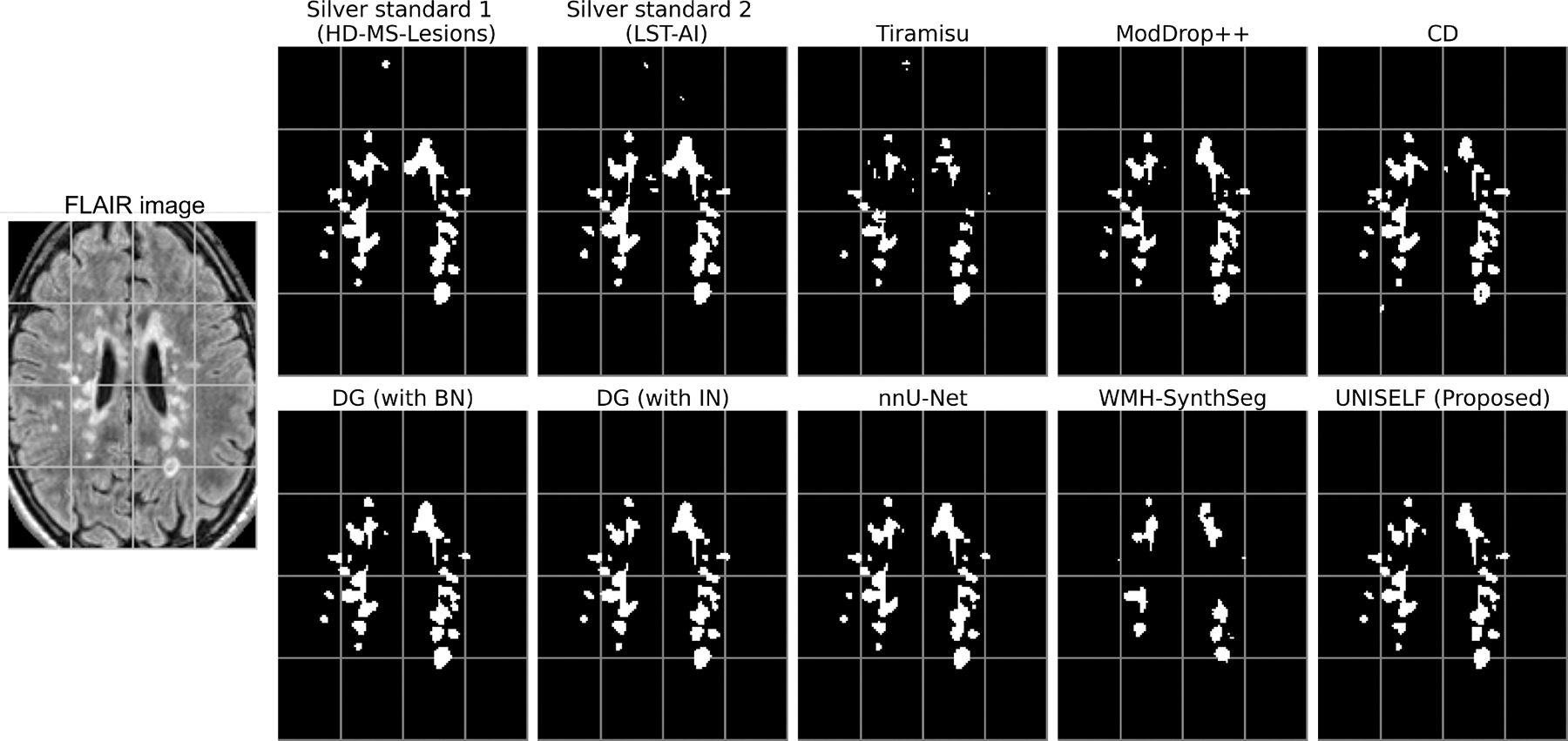
MS lesion segmentation masks from different methods on a representative subject in the private test dataset. “UNISELF (Proposed)” refers to the finalized UNISELF configuration selected through cross-validation, as described in Section 4.3.

Figs. 5, S2, and S3 show MS lesion masks predicted by different methods on a representative subject from the private test dataset, demonstrating segmentation on the original contrast (Fig. 5), FLAIR with motion artifact (Fig. S2), and input with missing FLAIR (Fig. S3). In Fig. 5, missed lesions were observed in the benchmark methods *Tiramisu*, *CD* (contrast dropout), and *WMH-SynthSeg*. In contrast, the benchmark methods *DG* (domain generalization) with BN and IN, the benchmark method *nnU-Net*, and the proposed method *UNISELF* showed very few missed lesions. In Fig. S2, with motion artifacts added to the FLAIR image, more missed lesions were observed in the benchmark methods *Tiramisu*, *ModDrop*+ +, *CD*, and *WMH-SynthSeg*, but were not observed in *DG* with BN and IN, *nnU-Net*, and *UNISELF*. In Fig. S3, only *UNISELF* and the benchmark method *DG* with IN demonstrated robust segmentation performance when dealing with missing FLAIR.

**Fig. 6.**
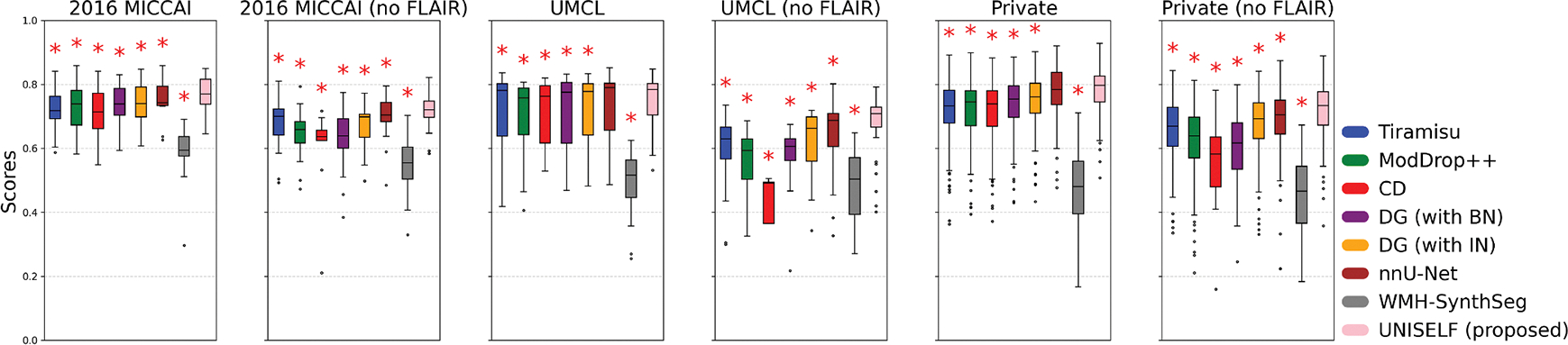
Segmentation scores (Eq. (5)) by different methods on public and private test datasets considering both original and missing FLAIR multicontrast inputs. “UNISELF (Proposed)” refers to the finalized UNISELF configuration selected through cross-validation, as described in Section 4.3. (Red star: statistically significant difference compared to UNISELF (Proposed) in each boxplot, based on the paired Wilcoxon signed-rank test with FDR-BH correction, *p* < .05.).

**Fig. 7.**
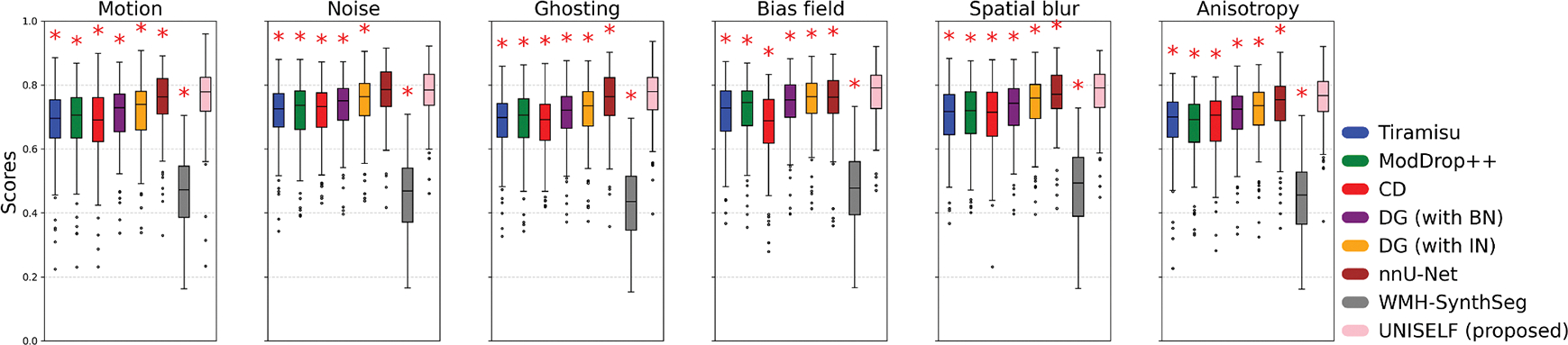
Segmentation scores (Eq. (5)) by different methods on the private multisite test dataset with various FLAIR artifacts. “UNISELF (Proposed)” refers to the finalized UNISELF configuration selected through cross-validation, as described in Section 4.3. (Red star: statistically significant difference compared to UNISELF (Proposed) in each boxplot, based on the paired Wilcoxon signed-rank test with FDR-BH correction, *p* < .05.).

Fig. 6 shows boxplots of segmentation scores (Eq. (5)) across Test Datasets II, III, and IV for the original multicontrast inputs and the corresponding inputs without FLAIR. *UNISELF* achieved noticeable improvements compared to the benchmark methods, especially when the FLAIR contrast was missing. Fig. 7 shows boxplots of segmentation scores (Eq. (5)) across Test Dataset IV with various artifacts added to the FLAIR contrast. The benchmark methods *DG* with IN and BN had already been exposed to such artifacts during training through DG augmentation. Nevertheless, *UNISELF* outperformed all benchmark methods, including *DG* with BN and IN. Individual metrics of all methods across all out-of-domain tests are provided in Supplementary Tables S1–S12, where considerably lower DSC and TPR values (indicating under-segmentation) and higher LFPR values (reflecting a much smaller number of predicted lesions, as used in the denominator of LFPR) were observed in *Tiramisu*, *ModDrop*+ +, *CD*, and *WMH-SynthSeg*.

## 6. Method analysis

### 6.1. Ablation study

We conducted an ablation study on the two key components from Sections 3.2 and 3.3, using both 2.5D (as implemented in the proposed *UNISELF*) and 3D U-Net architectures. All models in this ablation study were trained with CD to ensure consistent training conditions across architectures and configurations. The study systematically examined the impact of removing these components: (1) replacing self-ensembled lesion fusion (Section 3.2) with conventional three-plane majority voting, and (2) replacing TTIN (trained with BN and CondIN) (Section 3.3) with BN training statistics. The 3D U-Net architecture includes patch-based sliding-window processing, as implemented in *nnU-Net* (Isensee et al., 2021), using a patch size of 112 × 112 × 112 and a step size of 56 × 56 × 56. The parameters $\tau_{1}$ and $\tau_{2}$ used in the self-ensembled lesion fusion for 3D architectures were selected through cross-validation on the ISBI training set (consistent with the procedure described in Section 4.3), with $\tau_{1}=22$ and $\tau_{2}=14$ for BN training statistics, $\tau_{1}=20$ and $\tau_{2}=7$ for TTIN trained with BN, and $\tau_{1}=17$ and $\tau_{2}=10$ for TTIN trained with CondIN. Figures S4 and S5 show boxplots of segmentation scores (Eq. (5)) from the ablation study, corresponding to the same out-of-domain experiments shown in Figs. 6 and 7. For both 3D and 2.5D U-Net architectures, adding self-ensembled lesion fusion and TTIN (trained with BN and CondIN) progressively improved their segmentation scores, especially in Fig. S5, which shows results on Test Dataset IV with various artifacts added to the FLAIR contrast. Moreover, the 2.5D U-Net (as implemented in the proposed *UNISELF*) generally outperformed the 3D U-Net across all out-of-domain experiments.

### 6.2. Visualization of latent features normalized by TTIN (trained with BN)

**Fig. 8.**
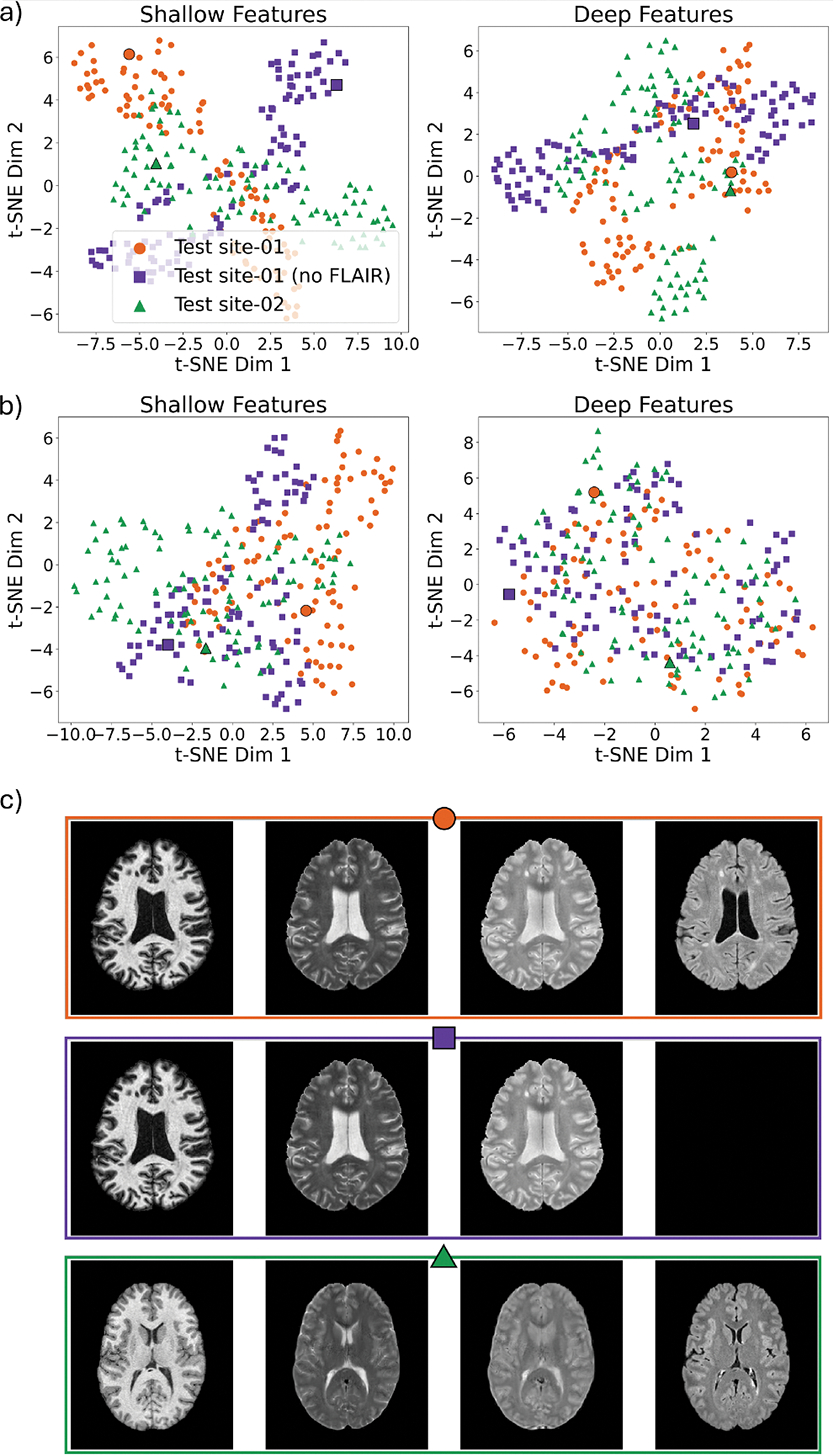
t-SNE visualization of feature statistics across three test datasets. (a): t-SNE of normalized features in shallow (left) and deep (right) layer features using training dataset normalization statistics. (b): Corresponding t-SNE using instance-specific normalization statistics at test-time. (c): Example multicontrast images (represented by larger markers in (a) and (b)) from the three test datasets.

Fig. 8 presents t-SNE plots of U-Net’s (trained with CD and BN) shallow (highest level in the encoder path) and deep (lowest level in the encoder path) layer features across three test sites. In this visualization, t-SNE was applied to the concatenated channel-wise mean and variance of the normalized features. The three test sites include: ISBI challenge test dataset (‘Site-01’), a test site from Test Dataset IV (‘Site-02’), and a modified version of the ISBI challenge test dataset where the FLAIR contrast was replaced with zeros (‘Site-01 (no FLAIR)’). Ten axial slices, spaced 10 slices apart, were extracted from each of the 10 subjects at each test site, with each dot in Fig. 8 representing one of these inputs. Fig. 8a shows t-SNE plots of features normalized using the training dataset normalization statistics $E_{MA}\left[x_{j}\right]$ and ${Var}_{MA}\left[x_{j}\right]$ of the corresponding layer (Ioffe and Szegedy, 2015). As a comparison, Fig. 8b shows t-SNE plots of the same features with TTIN (trained with BN) applied. Fig. 8c shows representative multicontrast slices from each test site, including full multicontrast images (first row), as well as cases with missing FLAIR (second row) and contrast variations (third row). Separated clusters from each test site were observed in both shallow and deep layers in Fig. 8a, indicating mismatched features across different test sites. However, this mismatch was not observed after applying TTIN (trained with BN), as shown in Fig. 8b.

### 6.3. Robustness of $\tau_{1}$ and $\tau_{2}$ parameters in the ISBI test

**Fig. 9.**
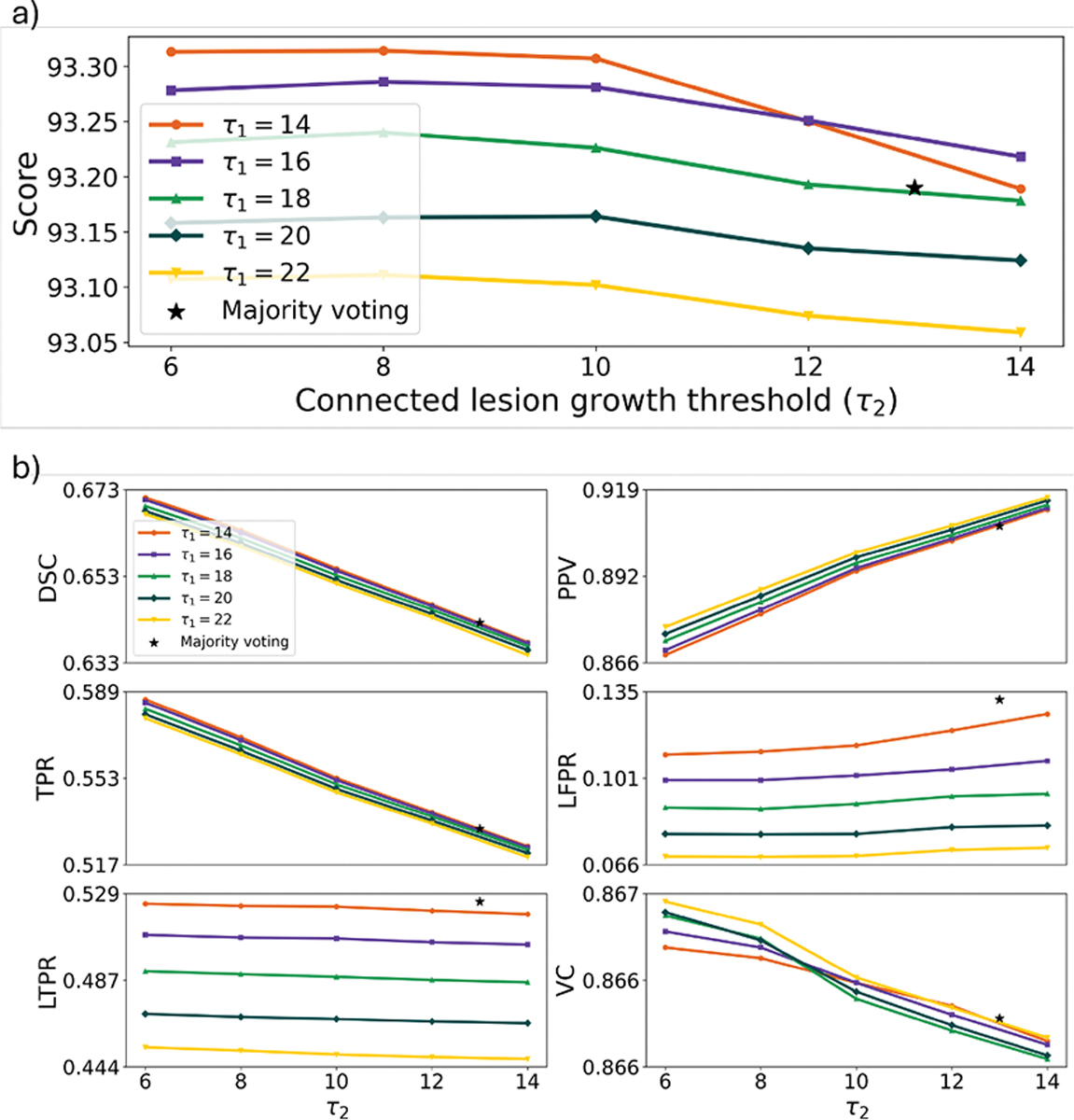
(a): The robustness of the lesion detection $\tau_{1}$ (in Eq. (2)) and connected lesion growth $\tau_{2}$ (in Eq. (3)) parameters on the segmentation score (Eq. (5)) in the ISBI challenge test. (b): The impact of $\tau_{1}$ and $\tau_{2}$ on individual metrics. For comparison, segmentation metrics from majority voting ($\tau_{1}=\tau_{2}=13$, marked with stars) are also presented.

In addition to the main test experiments in Sections 4.4 and 4.5 using the finalized *UNISELF*, we conducted separate comparative analyses in Sections 6.3 thru 6.5 on both in-domain and out-of-domain test datasets, evaluating different configurations of the self-ensembled lesion fusion (Section 3.2) and TTIN variants (Section 3.3) components. These analyses were motivated by the consistently strong cross-validation performance (Fig. S1) observed across varying $\tau_{1}$ and $\tau_{2}$ values (Eqs. 2 and 3) and different normalization strategies for learning $\gamma_{j}$ and $\beta_{j}$ (Eq. (4b)). A robustness analysis of the lesion detection $\tau_{1}$ (in Eq. (2)) and connected lesion growth $\tau_{2}$ (in Eq. (3)) parameters was conducted to evaluate their impact on segmentation performance using the ISBI challenge test dataset. Fig. 9 illustrates the impact of $\tau_{1}$ and $\tau_{2}$ on the overall segmentation score (Eq. (5)) and on individual metrics. In Fig. 9a, we observe that an optimal score of 93.32 was achieved with $\tau_{1}=14$ and $\tau_{2}=8$. Consistent scores above 93.20 were achieved for $14\leq \tau_{1}\leq 18$ and $6\leq \tau_{2}\leq 12$, outperforming the majority voting ( $\tau_{1}=\tau_{2}=13$ ) score of 93.19. Fig. 9b shows the effect of $\tau_{1}$ and $\tau_{2}$ on individual metrics. In Fig. 9b, we observe that the change in $\tau_{1}$ at each fixed $\tau_{2}$ has relatively little impact on DSC, PPV and TPR, while larger values of $\tau_{2}$ contribute to improved PPV at the expense of decreased DSC and TPR. However, LFPR and LTPR were primarily affected by $\tau_{1}$, with higher values of $\tau_{1}$ resulting in improved LFPR but worse LTPR. Notably, only very small variations in VC are observed across different $\tau_{1}$ and $\tau_{2}$ values.

### 6.4. Impact of different TTIN variants on CD performance in the ISBI test

For the ISBI test dataset, we conducted a comprehensive experiment using all input contrast combinations and investigated the impact of different TTIN variants on models trained with and without CD. In this experiment, TTIN variants trained with BN, IN, and CondIN were compared with the use of BN training dataset statistics for feature normalization at test-time, as originally proposed in BN (Ioffe and Szegedy, 2015). TTIN with various feature normalization methods for training $\gamma_{j}$ and $\beta_{j}$ in Eq. (4b) was also employed and compared, including BN, IN, and CondIN as described in Section 3.3. The proposed self-ensemble described in Section 3.2 was applied to all models, with specific $\tau_{1}$ and $\tau_{2}$ values corresponding to each configuration provided in Table S13. Statistical significance (*p* < .05) was assessed using the paired Wilcoxon signed-rank test over 122 subject-level average scores (61 test subjects evaluated against two expert rater references), with each score computed by averaging performance across all 15 input contrast combinations.

First, our results (see Table S13) show that the performance was consistently higher in independently trained models without CD for each input contrast combination compared to those with CD, especially when the FLAIR contrast was removed from the input. Second, for models trained with BN, switching from BN training statistics to TTIN (trained with BN) statistics at test-time improved the performance of CD-trained models (*p* < .05 comparing the second and fourth columns in Table S13). Specifically, this resulted in a noticeable improvement in test scores with TTIN (trained with BN) statistics (fourth column in Table S13) compared to BN training statistics (second column in Table S13) for input contrast combinations (rows) highlighted by a teal-to-gold color-shaded value change. Furthermore, no significant differences were observed among CD-trained TTIN models using BN, CondIN, and IN (*p* > .05 for all paired comparisons among the three models). For complete segmentation scores in this experiment, see Table S13.

### 6.5. Impact of different TTIN variants on CD performance in out-of-domain tests

We also conducted experiments to investigate the impact of different TTIN variants (trained with BN, IN, and CondIN) on models trained with and without CD using Test Datasets II, III, and IV. The proposed self-ensemble strategy (Section 3.2) was applied to all models. Results (see Tables S14 and S15) show that when different TTIN variants (trained with BN, IN, and CondIN) was employed, CD-trained models consistently outperformed independently trained models without CD across all test datasets. However, when BN training statistics were employed, CD-trained models showed degraded performance compared to independently trained models when the FLAIR contrast was missing. Comparable and optimal performance was observed on *UNISELF* trained with BN, IN, and CondIN. For the complete segmentation scores with statistical significance tests in these experiments, please refer to Tables S14 and S15.

**Fig. 10.**
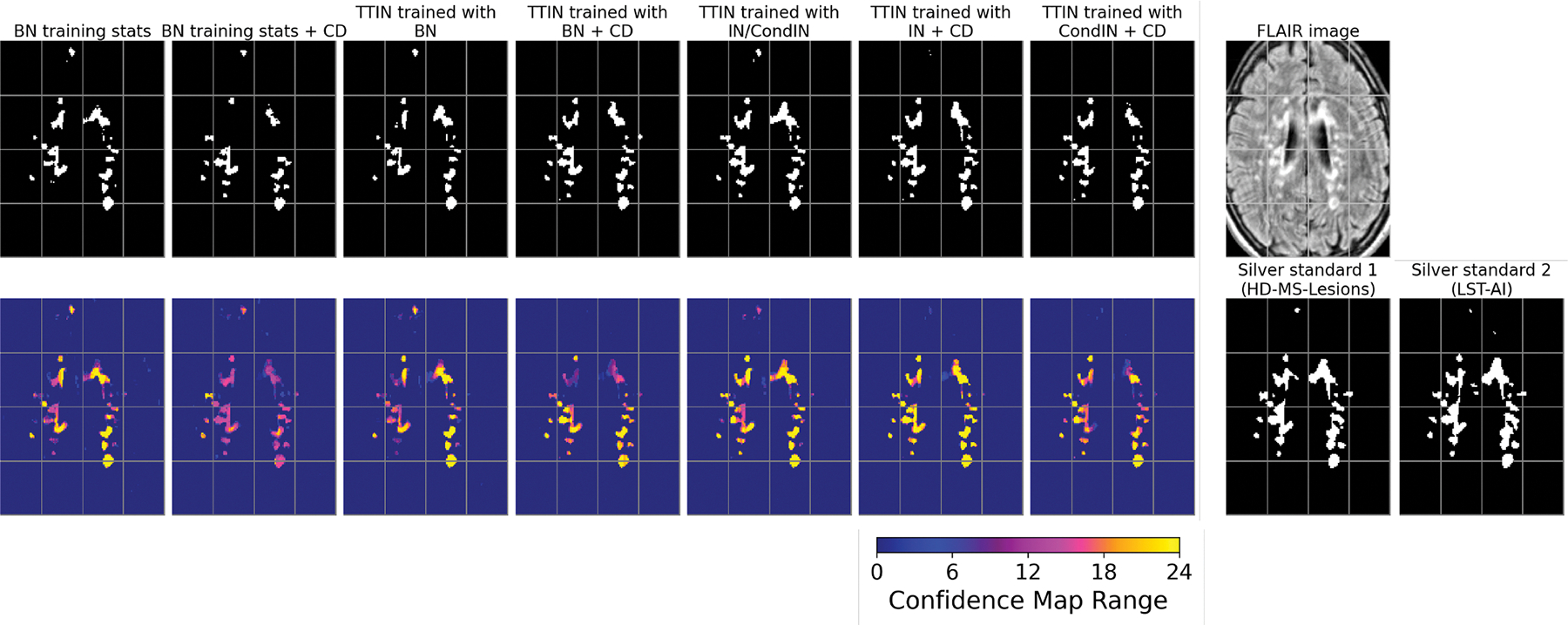
Impact of different TTIN variants (Section 3.3) on MS lesion segmentation masks comparing models trained with and without contrast dropout (CD). The same subject with FLAIR motion artifacts as depicted in Fig. S2 is presented.

Fig. 10 shows segmentation results and their corresponding confidence maps (Eq. (1)) for the methods listed in Table S15 on a representative subject from the private test dataset with FLAIR motion artifacts. Models using BN training statistics, both with and without CD, exhibited under-segmentation of lesions (columns 1–2). Moreover, TTIN trained with BN but without CD also exhibited noticeable under-segmentation of lesions (column 3). Applying CD resolved the under-segmentation issue (column 4). Similarly, no noticeable missing lesions were observed in TTIN trained with IN and CondIN when CD was applied (columns 6–7).

## 7. Discussion

### 7.1. Self-ensembled lesion fusion improves segmentation accuracy

UNISELF employs a novel self-ensembled lesion fusion strategy (Section 3.2) for TTA to improve segmentation accuracy. The proposed two-step lesion fusion strategy outperformed the conventional single-step voting approaches (majority-voting results in Figs. 9, S4 and S5) commonly used in prior methods (Isensee et al., 2021; Henschel et al., 2020). Unlike conventional TTA averaging or majority voting, which simply aggregates multiple predictions voxel-wise using a single threshold, the proposed self-ensembled lesion fusion explicitly separates lesion *detection* and *connected growth* through two thresholds $\left(\tau_{1},\tau_{2}\right)$, forming a structured two-threshold process. In UNISELF, the self-ensemble parameters $\tau_{1}$ and $\tau_{2}$ primarily affect lesion-wise and voxel-wise metrics, respectively (Fig. 9b). A possible explanation is that voxel-wise metrics such as DSC, PPV, and TPR are more susceptible to the total volume of segmented lesions, primarily influenced by $\tau_{2}$. In contrast, lesion-wise metrics such as LFPR and LTPR are largely determined by lesion detection, which $\tau_{1}$ mainly affects. This concept of “detection and growth” using high and low threshold values was also proposed and proven effective in Canny edge detection (Canny, 1986) with Gaussian-filtered edge detection to generate candidate edges before thresholding. Compared to Canny edge detection, UNISELF generates candidate lesions (C(r) in Fig. 2a) through its comprehensive multi-orientation processing of 3D multicontrast MR volumes (Fig. 1) using a unified segmentation model.

### 7.2. TTIN enhances generalization to varying contrasts

UNISELF leverages TTIN (trained with CondIN) (Section 3.3) to improve model generalization handling missing contrasts. The effectiveness of different TTIN variants (trained with BN, IN, and CondIN) in addressing varying contrast combinations in CD-trained models was validated using the ISBI challenge test data (Table S13). Simply switching to TTIN stats after BN training, as indicated by ‘TTIN trained with BN’ in Table S13, significantly improves the performance of CD-trained models. This indicates that when training a lesion segmentation model with CD, sharing BN feature statistics among contrast combinations is not sufficient for optimal performance across all combinations. There is a tendency for these statistics to favor combinations that include FLAIR, thus sacrificing the performance of combinations without FLAIR, which represent a more challenging condition as evidenced by the markedly lower scores for FLAIR-missing combinations in Table S13. In contrast, TTIN trained with BN, IN, and CondIN avoid this issue by applying instance-specific feature normalization to each input combination, thereby achieving performance comparable to their independently trained counterparts. Unlike imputation-based methods, which attempt to synthesize missing contrasts through image translation models that often require complex training pipelines and risk hallucinated or low-fidelity content, UNISELF addresses missing contrast without explicit synthesis. Specifically, CD during training exposes the model to random contrast combinations, while different TTIN variants (e.g., TTIN trained with CondIN as used in UNISELF) recalibrate feature distributions at inference by updating normalization statistics for each contrast combination. This design enables effective generalization to real-world scenarios where ideal contrast combinations may not be available. For completeness, we evaluated different TTIN variants across all possible input contrast combinations to comprehensively assess model behavior under varying conditions. However, in clinical and research settings, only a subset of these conditions are practically relevant. For example, lesions can be segmented reasonably well using T1w and T2w images when FLAIR is unavailable, whereas T1w-only inputs are not sufficient for reliable lesion delineation.

### 7.3. Validation on out-of-domain and missing FLAIR scenarios

The impact of different TTIN variants on model generalization was further validated using various out-of-domain test datasets (Tables S14 and S15). For both CD-trained and independently trained models, inferior performance was observed in models using BN training stats compared to those using TTIN variants trained with BN, IN, or CondIN. This inferiority was more pronounced for CD-trained models when the FLAIR contrast was missing (‘No FLAIR’ in Table S14). In fact, CD training has been reported to improve domain generalization (Zhang et al., 2023a). Our results support this finding and further demonstrate that all three TTIN variants (trained with BN, IN, and CondIN) are effective for the implementation of CD training. FLAIR is well known to be an ideal contrast for MS diagnosis and monitoring (Wattjes et al., 2015), and manual lesion annotations are therefore primarily guided by FLAIR images (Carass et al., 2017b). Accordingly, the performance drop observed when FLAIR is missing is an expected outcome, and our results in Table S14 are consistent with this observation. Although FLAIR was introduced in the early 1990s and rapidly adopted due to its superior lesion conspicuity (Hajnal et al., 1992b,a), it began to be incorporated into routine clinical practice in the 2000s, with broader standardization for MS MRI protocols following the 2015 MAGNIMS consensus guidelines (Filippi et al., 2016). As a result, many legacy multicenter or longitudinal datasets collected in the 1990s-2000s lack FLAIR imaging and rely primarily on T2w and PDw sequences (Filippi et al., 1995; Comi et al., 1993; Katz et al., 1993).

### 7.4. Comparison with other test-time adaptation strategies

Prior work has explored test-time strategies to address distribution shifts. Nado et al. (2020) demonstrated that recomputing BN statistics at prediction time for each test batch improves model robustness under domain shift. Gérin et al. (2024) applied test-time training (TTT) with auxiliary tasks on a per-patient basis to mitigate domain shifts without requiring data from other patients. In comparison, we adopt TTIN trained with CondIN in UNISELF, and all TTIN variants trained with BN, IN, and CondIN offer several advantages for MS lesion segmentation. First, unlike prediction-time BN, TTIN operates per test input without batch size dependency, making it directly applicable on-the-fly in clinical workflows. Second, unlike TTT, TTIN is lightweight and training-free, requiring no additional model updates. Third and most importantly, we show that TTIN is especially beneficial for CD-trained models, as it mitigates intrinsic distribution shifts from varying contrast combinations even when the model is exposed to all combinations during training. Our work complements recent benchmarking efforts on domain shift (Malinin et al., 2022), demonstrating that TTIN offers a practical and robust alternative to TTT and prediction-time BN in MS lesion segmentation.

### 7.5. Generalization without large-scale annotated data

State-of-the-art MS lesion segmentation methods trained on large and heterogeneous datasets, such as HD-MS-Lesions (Brugnara et al., 2020) and LST-AI (Wiltgen et al., 2024), represent another strategy for improving model generalization. These methods benefit from broad data diversity and extensive expert annotations, which help capture intersite and inter-scanner variations. However, such large-scale annotated datasets are often not publicly available. In contrast, our method is designed for scenarios where access to large heterogeneous datasets is not feasible and where protocol variability and missing contrasts are major concerns. By leveraging self-ensembled lesion fusion and TTIN trained with CondIN, UNISELF achieves strong generalization performance under limited training data and in the presence of domain shifts or missing MRI contrasts.

### 7.6. Broader applications

In addition to MS lesion segmentation, there is growing interest in developing automated detection methods for paramagnetic rim lesions with lesion-level analysis in MS (Zhang et al., 2022; Barquero et al., 2020; Lou et al., 2021). UNISELF’s detection-and-connected-growth component may be applied to identify distinct lesions by separating confluent lesions (Dworkin et al., 2018; Wynen et al., 2025; Rivas et al., 2025), enabling more accurate lesion-wise analyses relevant to rim lesions. In addition, the proposed self-ensemble strategy and test-time instance normalization in UNISELF may also be extended to rim lesion detection itself, for example by aggregating predictions across multi-orientation inputs using a tunable threshold similar to $\tau_{1}$ in UNISELF, with rigorous multi-site validation. Beyond downstream MS segmentation and rim lesion detection, there has been active research in developing cross-domain generalizable deep learning models for low-level image formation tasks with limited training data. For instance, there has been increased interest in enhancing the generalization capacity of MRI reconstruction models trained on single-vendor data, particularly against deviations in acquisition parameters and vendor changes (Muckley et al., 2021; Zhang et al., 2023b). This improvement is facilitated by incorporating a self-adaptive k-space data consistency module within the networks. Other examples include test-time domain adaptation to unseen domains, which is achieved using data consistency loss (Zhang et al., 2020a,b; Yoo et al., 2021; Güngör et al., 2023). UNISELF for MS lesion segmentation resembles self-adaptive data consistency for MRI reconstruction in terms of its full exploitation of the input data through self-ensemble and self-adaptive latent features through TTIN trained with CondIN. Notably, self-ensemble can also be applied to MRI reconstruction by averaging reconstructions from rotated and flipped k-space data and leveraging their variance to assess reconstruction uncertainty.

### 7.7. Limitations and improvement

There are some limitations of this work. First, the UNISELF model from the final training epoch was selected for inference, which may introduce a potential risk of overfitting. Future work will consider incorporating *k*-fold cross-validation on the training dataset to mitigate this risk. Second, the two thresholds $\tau_{1}$ and $\tau_{2}$ are fixed across all locations in the brain, which may not be optimal for detecting and segmenting certain types of lesions in MS, such as small cortical lesions (La Rosa et al., 2020). Implementing spatially adaptive thresholds with lower $\tau_{1}$ and $\tau_{2}$ values near the cortical regions may improve the sensitivity of cortical lesion detection and segmentation. Third, although processing multi-orientation inputs using a single network (Fig. 1) is advantageous for implementation simplicity in UNISELF, exploring advanced architectures for more effective multi-orientation processing is still needed. The Mixture-of-Experts (MoE) architecture (Jacobs et al., 1991) is an option that treats multi-orientation inputs as subproblems to form an expert-guided pathway for a given input data. Fourth, although the reference delineations generated for the private multi-site dataset were obtained using automated tools (LST-AI and HD-MS-Lesions) and were carefully crafted, they were still “silver-standard” annotations and not as convincing as the expert delineations available in other test datasets. Finally, while UNISELF demonstrated good generalization when trained with limited single-site labeled data, its generalization may be further improved by leveraging large-scale unlabeled clinical multicontrast MRI datasets through self-supervised pretraining (Tang et al., 2022) or by training with synthetic MRI containing MS lesions (Zhang et al., 2025).

## 8. Conclusion

In conclusion, we propose UNISELF, a method that achieves state-of-the-art MS lesion segmentation both in-domain and out-of-domain under the constraints of training on the limited single-source ISBI training dataset with missing contrasts at inference. By integrating a novel self-ensembled lesion fusion strategy and leveraging test-time instance normalization, UNISELF ranks among the best-performing methods on the ISBI challenge test set and demonstrates superior generalization to out-of-domain datasets with contrast variation, imaging artifacts, and missing contrasts.

## Supplementary Material

1

Supplementary material associated with this article can be found in the online version at 10.1016/j.media.2026.103954.

## Figures and Tables

**Table 1:** Performance comparison on the ISBI 2015 Challenge test data. ‘UNISELF (Proposed)” denotes the final configuration selected via cross-validation. Statistical significance was assessed using the paired Wilcoxon signed-rank test with FDR-BH correction (*p* < .05).

Method	Score (↑)	DSC (↑)	PPV (↑)	TPR (↑)	LFPR (↓)	LTPR (↑)	VC (↑)

ALL-Net (in paper) (Zhang et al., 2021a)	**93.320**	0.639	**0.914**	0.525	0.122	0.533	0.860
Tiramisu (in paper) (Zhang et al., 2019a)	93.210	0.643	0.908	0.533	0.124	0.520	**0.867**

Tiramisu (Zhang et al., 2019a)	92.433 ± 8.016*	0.668 ± 0.136*	0.860 ± 0.109*	0.585 ± 0.200*	0.279 ± 0.164*	**0.586 ± 0.231** *	0.860
ModDrop++ (Liu et al., 2022)	92.296 ± 8.709*	0.668 ± 0.133*	0.859 ± 0.108*	0.582 ± 0.195*	0.291 ± 0.168*	0.580 ± 0.231*	0.863
CD (Feng et al., 2019)	92.394 ± 8.568*	0.672 ± 0.130 *	0.839 ± 0.107*	0.598 ± 0.195 *	0.235 ± 0.158*	0.544 ± 0.233*	0.863
DG (with BN) (Zhang et al., 2023a)	91.914 ± 8.696*	0.659 ± 0.130	0.829 ± 0.109*	0.586 ± 0.195*	0.324 ± 0.165*	0.583 ± 0.231 *	0.860
DG (with IN) (Zhang et al., 2023a)	92.427 ± 8.210*	0.650 ± 0.138*	0.858 ± 0.098*	0.557 ± 0.193*	0.246 ± 0.152*	0.562 ± 0.231*	0.862
nnU-Net (Isensee et al., 2021)	92.882 ± 7.962*	**0.684 ± 0.135** *	0.840 ± 0.120*	**0.618 ± 0.203** *	0.149 ± 0.132*	0.516 ± 0.230*	0.863
WMH-SynthSeg (Laso et al., 2024)	85.875 ± 18.093*	0.366 ± 0.136*	0.584 ± 0.211*	0.300 ± 0.140*	0.314 ± 0.235*	0.172 ± 0.145*	0.605
UNISELF (Proposed)	93.286 ± 7.400	0.664 ± 0.141	0.882 ± 0.099	0.569 ± 0.199	**0.100 ± 0.102**	0.508 ± 0.230	0.866

(Top two rows: average performance metrics directly taken from original publications of ALL-Net and Tiramisu; subject-level results are unavailable. Remaining rows: mean and standard deviation of performance metrics for benchmark models and UNISELF (Proposed) trained in-house (Section 4.2), all obtained from official ISBI submissions with subject-level results.

* : statistically significant difference compared to UNISELF (Proposed) in each column, based on paired Wilcoxon signed-rank test with FDR-BH correction (*p* < .05). The best and second-best performances in each column are shown in **bold** and underline, respectively.)

**Table 2:** Performance comparison on the ISBI 2015 Challenge test data (no FLAIR). ‘UNISELF (Proposed)” denotes the final configuration selected via cross-validation. Statistical significance was assessed using the paired Wilcoxon signed-rank test with FDR-BH correction (*p* < .05).

Method	Score (↑)	DSC (↑)	PPV (↑)	TPR (↑)	LFPR (↓)	LTPR (↑)	VC (↑)

Tiramisu (Zhang et al., 2019a)	91.897 ± 8.276*	0.559 ± 0.151*	0.852 ± 0.091*	0.446 ± 0.181*	0.232 ± 0.155*	0.529 ± 0.221*	0.857
ModDrop++ (Liu et al., 2022)	90.799 ± 9.915*	0.521 ± 0.160*	0.817 ± 0.091*	0.412 ± 0.182*	0.278 ± 0.169*	0.464 ± 0.208	0.861
CD (Feng et al., 2019)	89.064 ± 15.173*	0.189 ± 0.116*	0.932 ± 0.178*	0.110 ± 0.075*	0.054 ± 0.188*	0.174 ± 0.115*	0.751
DG (with BN) (Zhang et al., 2023a)	90.221 ± 11.830*	0.484 ± 0.150*	0.738 ± 0.150*	0.378 ± 0.155*	0.241 ± 0.173*	0.404 ± 0.201*	**0.871**
DG (with IN) (Zhang et al., 2023a)	90.992 ± 9.434*	0.564 ± 0.139*	0.756 ± 0.101*	0.479 ± 0.180*	0.326 ± 0.175*	0.543 ± 0.222*	0.868
nnU-Net (Isensee et al., 2021)	92.085 ± 8.061*	0.592 ± 0.140*	0.846 ± 0.097	0.486 ± 0.183*	0.135 ± 0.126	0.442 ± 0.205*	0.860
WMH-SynthSeg (Laso et al., 2024)	85.883 ± 15.259*	0.348 ± 0.129*	0.555 ± 0.214*	0.280 ± 0.122*	0.388 ± 0.214*	0.188 ± 0.143*	0.771
UNISELF (Proposed)	**92.386 ± 7.238**	0.574 ± 0.153	0.850 ± 0.079	0.468 ± 0.190	0.113 ± 0.108	0.462 ± 0.213	0.870

(Mean and standard deviation of performance metrics for benchmark models and UNISELF (Proposed) trained in-house (Section 4.2), all obtained from official ISBI submissions with subject-level results.

* : statistically significant difference compared to UNISELF (Proposed) in each column, based on paired Wilcoxon signed-rank test with FDR-BH correction (*p* < .05). The best and second-best performances in each column are shown in **bold** and underline, respectively.)

## Data Availability

Public datasets, including 2015 ISBI challenge, 2016 MICCAI challenge, and UMCL, are open-source and publicly available, whereas the private dataset remains confidential and will not be shared.

## Footnotes

1
https://mipav.cit.nih.gov/

2
https://www.nitrc.org/projects/toads-cruise/

3
https://stnava.github.io/ANTs/

4
https://surfer.nmr.mgh.harvard.edu/fswiki/WMH-SynthSeg/
