# Supplementary material for: UNISELF: A unified network with instance normalization and self-ensembled lesion fusion for multiple sclerosis lesion segmentation
Source: Med Image Anal. Author manuscript; Available in PMC 2026 Mar 2. (PMC12951640; doi:10.1016/j.media.2026.103954)
Supplement: 1 [file NIHMS2140940-supplement-1.pdf]

Supplementary Material

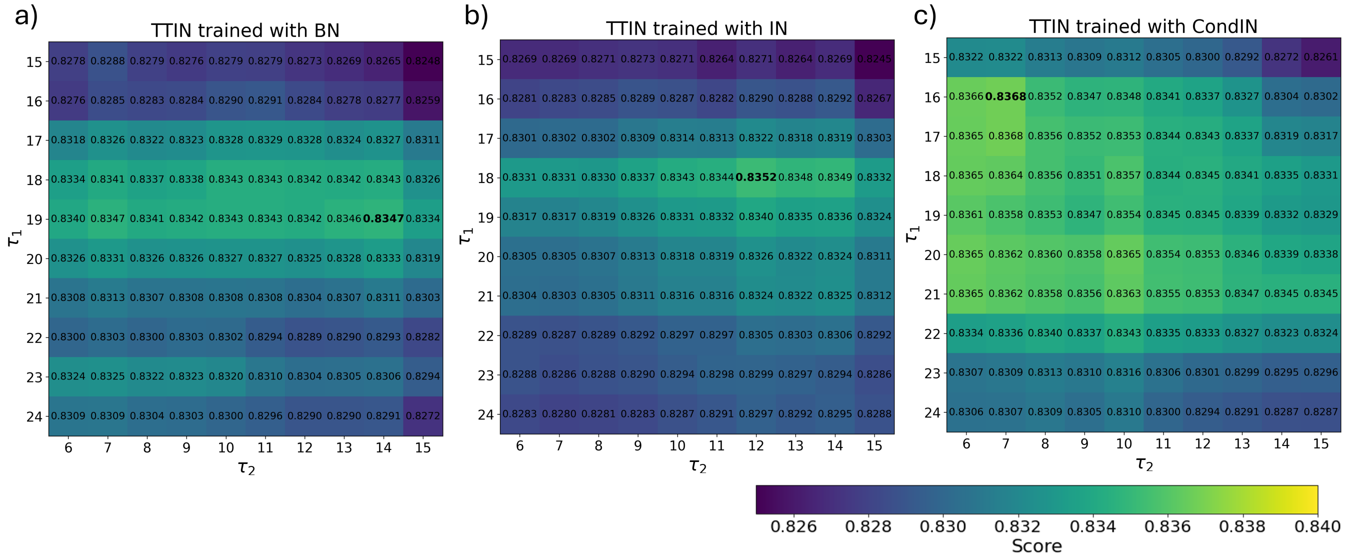

Fig. S1. Cross-validation grid search of self-ensembled lesion fusion parameters τ_1 and τ_2 for models employing TTIN trained with BN, IN, and CondIN. Each heatmap shows the average validation score on the ISBI training set using five-fold cross-validation, with one subject held out for validation in each fold. Scores are computed for each (τ_1, τ_2) pair, and bolded values indicate the highest performance within each (τ_1, τ_2) grid.

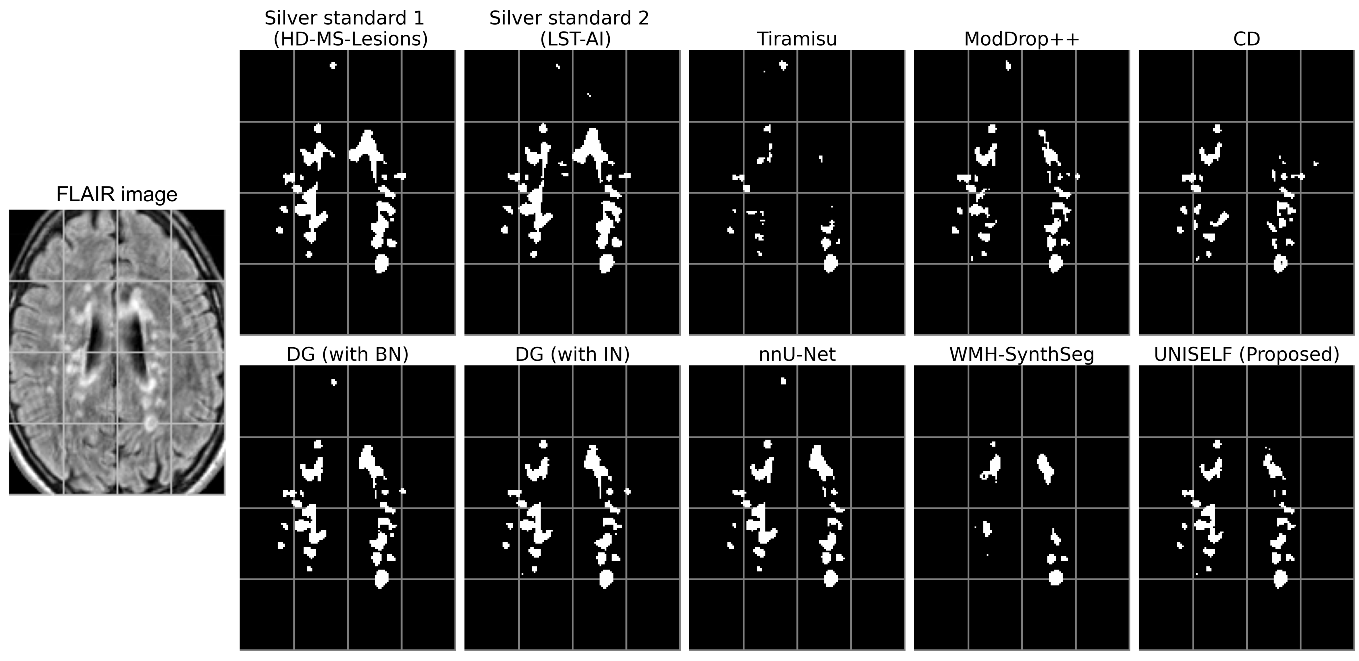

Fig. S2. MS lesion segmentation masks from different methods on the same subject as in Fig. 5, with motion artifacts present on the FLAIR image.

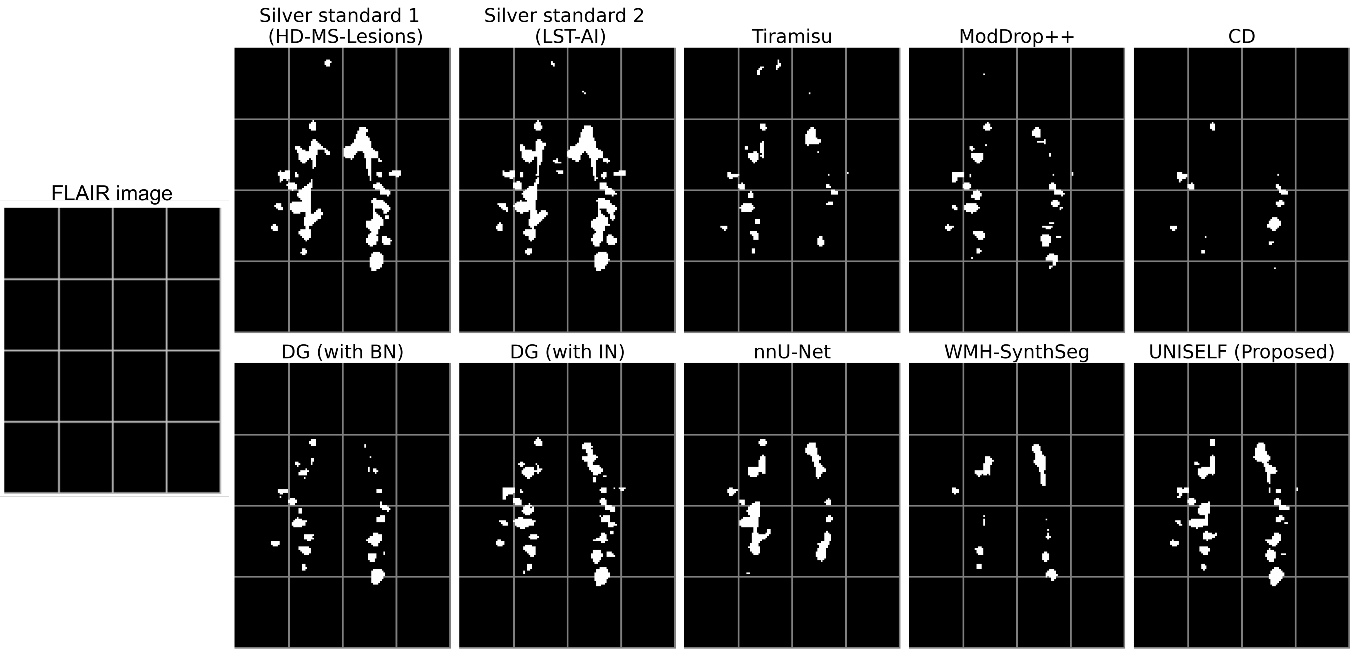

Fig. S3. MS lesion segmentation masks from different methods on the same subject as in Figs. 5 and S2, with missing FLAIR contrast.

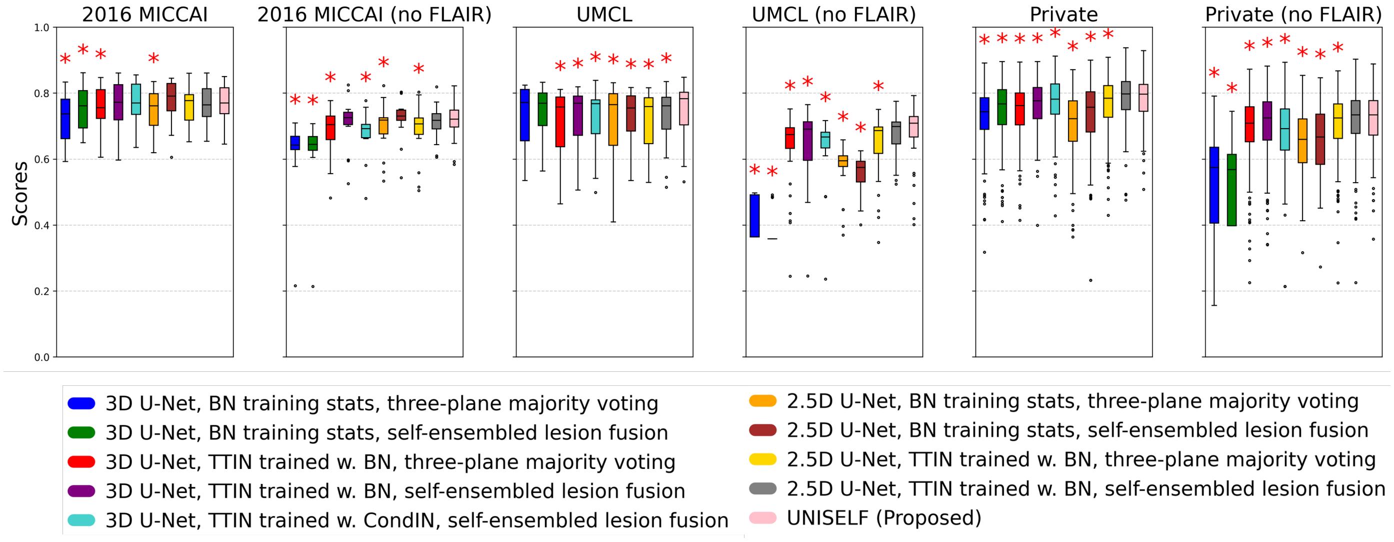

Fig. S4. Segmentation scores (Eq. (5)) from the ablation study on public and private test datasets, considering both original and missing FLAIR multicontrast inputs. Both 2.5D (as proposed) and 3D U-Net architectures were evaluated by progressively 1) replacing "self-ensembled lesion fusion" with "three-plane majority voting" and 2) replacing "TTIN trained with BN" with "BN training stats". "UNISELF (Proposed)" refers to the UNISELF configuration finalized in cross-validation (Section 4.3), which uses TTIN trained with CondIN. (Red star: statistically significant difference compared to UNISELF (Proposed) in each boxplot, based on the paired Wilcoxon signed-rank test with FDR-BH correction, $p < 0.05$.)

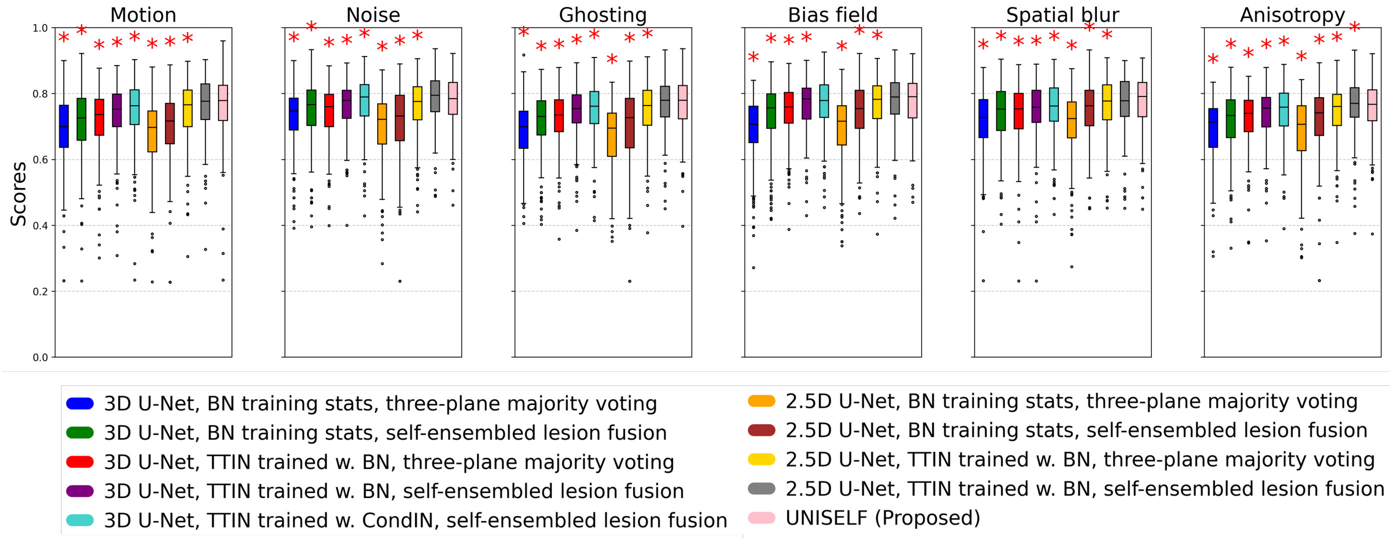

Fig. S5. Segmentation scores (Eq. (5)) from the ablation study on the private multisite test dataset containing various FLAIR artifacts. The same ablation procedure as in Fig. S4 was applied. (Red star: statistically significant difference compared to UNISELF (Proposed) in each boxplot, based on the paired Wilcoxon signed-rank test with FDR-BH correction, $p < 0.05$.)

Table S1. Performance comparison of segmentation methods on the MICCAI2016 dataset. "UNISELF (Proposed)" denotes the final configuration selected via cross-validation. Statistical significance was assessed using the paired Wilcoxon signed-rank test with FDR-BH correction ($p < 0.05$), excluding VC.

Method	Score (↑)	DSC (↑)	PPV (↑)	TPR (↑)	LFPR (↓)	LTPR (↑)	VC (↑)
Tiramisu (Zhang et al., 2019a)	0.722 ± 0.071*	0.664 ± 0.128	0.794 ± 0.144*	0.589 ± 0.151	0.407 ± 0.168*	0.591 ± 0.151*	0.975
ModDrop++ (Liu et al., 2022)	0.726 ± 0.076*	0.639 ± 0.132*	0.778 ± 0.151*	0.559 ± 0.153*	0.357 ± 0.164*	0.576 ± 0.164*	0.977
CD (Feng et al., 2019)	0.715 ± 0.081*	0.662 ± 0.120	0.751 ± 0.158*	0.607 ± 0.121	0.401 ± 0.178*	0.569 ± 0.159*	0.984
DG (with BN) (Zhang et al., 2023a)	0.732 ± 0.070*	0.674 ± 0.121	0.778 ± 0.147*	**0.608 ± 0.134***	0.429 ± 0.153*	**0.643 ± 0.142***	0.989
DG (with IN) (Zhang et al., 2023a)	0.736 ± 0.071*	0.670 ± 0.115	0.789 ± 0.140*	0.597 ± 0.134	0.417 ± 0.164*	0.641 ± 0.147*	0.989
nnU-Net (Isensee et al., 2021)	0.754 ± 0.061*	0.628 ± 0.114*	**0.847 ± 0.129***	0.516 ± 0.134*	0.196 ± 0.161	0.499 ± 0.194	0.974
WMH-SynthSeg (Laso et al., 2024)	0.587 ± 0.090*	0.381 ± 0.162*	0.507 ± 0.293*	0.341 ± 0.138*	0.329 ± 0.196*	0.293 ± 0.196*	0.941
UNISELF (Proposed)	**0.770 ± 0.057**	**0.678 ± 0.119**	0.818 ± 0.121	0.589 ± 0.136	**0.189 ± 0.108**	0.528 ± 0.174	**0.991**

(*: statistically significant difference compared to UNISELF (Proposed) in each column, based on paired Wilcoxon signed-rank test with FDR-BH correction ($p < 0.05$), excluding VC. The best and second-best performances in each column are shown in **bold** and underline, respectively.)

Table S2. Performance comparison of segmentation methods on the MICCAI2016 dataset (No FLAIR). "UNISELF (Proposed)" denotes the final configuration selected via cross-validation. Statistical significance was assessed using the paired Wilcoxon signed-rank test with FDR-BH correction ($p < 0.05$), excluding VC.

Method	Score (↑)	DSC (↑)	PPV (↑)	TPR (↑)	LFPR (↓)	LTPR (↑)	VC (↑)
Tiramisu (Zhang et al., 2019a)	0.677 ± 0.089*	0.523 ± 0.149*	0.805 ± 0.132	0.399 ± 0.144*	0.317 ± 0.171*	0.399 ± 0.198	0.964
ModDrop++ (Liu et al., 2022)	0.643 ± 0.082*	0.489 ± 0.155*	0.751 ± 0.182	0.378 ± 0.142*	0.335 ± 0.196*	0.341 ± 0.194*	0.946
CD (Feng et al., 2019)	0.612 ± 0.114*	0.286 ± 0.130*	**0.862 ± 0.236***	0.177 ± 0.092*	**0.141 ± 0.286**	0.173 ± 0.133*	0.843
DG (with BN) (Zhang et al., 2023a)	0.625 ± 0.102*	0.466 ± 0.171*	0.721 ± 0.209	0.359 ± 0.154*	0.347 ± 0.228*	0.312 ± 0.188*	0.941
DG (with IN) (Zhang et al., 2023a)	0.668 ± 0.075*	0.548 ± 0.153	0.727 ± 0.191*	**0.451 ± 0.147**	0.370 ± 0.161*	**0.445 ± 0.177***	0.959
nnU-Net (Isensee et al., 2021)	0.692 ± 0.074*	0.516 ± 0.110*	0.795 ± 0.139	0.389 ± 0.101*	0.182 ± 0.172	0.323 ± 0.191*	**0.971**
WMH-SynthSeg (Laso et al., 2024)	0.546 ± 0.092*	0.327 ± 0.171*	0.466 ± 0.290*	0.289 ± 0.137*	0.362 ± 0.241*	0.264 ± 0.161*	0.886
UNISELF (Proposed)	**0.715 ± 0.066**	**0.561 ± 0.139**	0.790 ± 0.146	0.450 ± 0.144	0.154 ± 0.124	0.383 ± 0.201	0.956

(*: statistically significant difference compared to UNISELF (Proposed) in each column, based on paired Wilcoxon signed-rank test with FDR-BH correction ($p < 0.05$), excluding VC. The best and second-best performances in each column are shown in **bold** and underline, respectively.)

Table S3. Performance comparison of segmentation methods on the UMCL dataset. "UNISELF (Proposed)" denotes the final configuration selected via cross-validation. Statistical significance was assessed using the paired Wilcoxon signed-rank test with FDR-BH correction ($p < 0.05$), excluding VC.

Method	Score (↑)	DSC (↑)	PPV (↑)	TPR (↑)	LFPR (↓)	LTPR (↑)	VC (↑)
Tiramisu (Zhang et al., 2019a)	0.722 ± 0.114*	0.629 ± 0.174*	0.812 ± 0.177	0.524 ± 0.161*	0.227 ± 0.221*	0.405 ± 0.117*	0.989
ModDrop++ (Liu et al., 2022)	0.708 ± 0.106*	0.638 ± 0.173*	0.791 ± 0.196*	0.549 ± 0.159	0.250 ± 0.213*	0.408 ± 0.103*	0.962
CD (Feng et al., 2019)	0.717 ± 0.093*	0.645 ± 0.150	0.806 ± 0.159*	0.545 ± 0.144	0.246 ± 0.186*	0.405 ± 0.116*	0.984
DG (with BN) (Zhang et al., 2023a)	0.719 ± 0.107*	0.659 ± 0.147	0.781 ± 0.186*	0.581 ± 0.132*	0.305 ± 0.219*	**0.484 ± 0.125***	0.979
DG (with IN) (Zhang et al., 2023a)	0.729 ± 0.101*	0.643 ± 0.158*	0.772 ± 0.206*	0.564 ± 0.137	0.212 ± 0.201*	0.443 ± 0.106*	0.978
nnU-Net (Isensee et al., 2021)	0.738 ± 0.098	**0.672 ± 0.159***	0.752 ± 0.217*	**0.621 ± 0.121***	0.136 ± 0.167*	0.382 ± 0.100*	**0.994**
WMH-SynthSeg (Laso et al., 2024)	0.495 ± 0.098*	0.310 ± 0.169*	0.523 ± 0.293*	0.252 ± 0.133*	0.257 ± 0.206*	0.129 ± 0.079*	0.690
UNISELF (Proposed)	**0.745 ± 0.085**	0.660 ± 0.150	**0.818 ± 0.177**	0.558 ± 0.139	**0.097 ± 0.128**	0.354 ± 0.113	0.984

(*: statistically significant difference compared to UNISELF (Proposed) in each column, based on paired Wilcoxon signed-rank test with FDR-BH correction ($p < 0.05$), excluding VC. The best and second-best performances in each column are shown in **bold** and underline, respectively.)

Table S4. Performance comparison of segmentation methods on the UMCL dataset (No FLAIR). "UNISELF (Proposed)" denotes the final configuration selected via cross-validation. Statistical significance was assessed using the paired Wilcoxon signed-rank test with FDR-BH correction ($p < 0.05$), excluding VC.

Method	Score (↑)	DSC (↑)	PPV (↑)	TPR (↑)	LFPR (↓)	LTPR (↑)	VC (↑)
Tiramisu (Zhang et al., 2019a)	0.594 ± 0.110*	0.419 ± 0.225*	0.676 ± 0.234*	0.323 ± 0.197	0.418 ± 0.196*	0.278 ± 0.115*	0.966
ModDrop++ (Liu et al., 2022)	0.559 ± 0.103*	0.247 ± 0.159*	0.664 ± 0.252*	0.161 ± 0.118*	0.287 ± 0.232*	0.192 ± 0.091	0.876
CD (Feng et al., 2019)	0.443 ± 0.064*	0.006 ± 0.018*	0.595 ± 0.486	0.003 ± 0.010*	**0.000***	0.011 ± 0.012*	0.460
DG (with BN) (Zhang et al., 2023a)	0.576 ± 0.090*	0.136 ± 0.129*	**0.760 ± 0.354**	0.080 ± 0.081*	0.086 ± 0.189	0.072 ± 0.075*	0.868
DG (with IN) (Zhang et al., 2023a)	0.623 ± 0.101*	0.437 ± 0.174	0.666 ± 0.207*	0.337 ± 0.156	0.319 ± 0.232*	**0.290 ± 0.110***	0.968
nnU-Net (Isensee et al., 2021)	0.644 ± 0.111*	**0.457 ± 0.185**	0.644 ± 0.236*	**0.361 ± 0.157***	0.162 ± 0.224*	0.207 ± 0.105	**0.979**
WMH-SynthSeg (Laso et al., 2024)	0.482 ± 0.103*	0.265 ± 0.148*	0.470 ± 0.284*	0.205 ± 0.106*	0.374 ± 0.241*	0.134 ± 0.081*	0.801
UNISELF (Proposed)	**0.668 ± 0.100**	0.442 ± 0.211	0.742 ± 0.225	0.329 ± 0.180	0.094 ± 0.167	0.196 ± 0.097	0.976

(*: statistically significant difference compared to UNISELF (Proposed) in each column, based on paired Wilcoxon signed-rank test with FDR-BH correction ($p < 0.05$), excluding VC. The best and second-best performances in each column are shown in **bold** and underline, respectively.)

Table S5. Performance comparison of segmentation methods on the private test dataset. "UNISELF (Proposed)" denotes the final configuration selected via cross-validation. Statistical significance assessed using paired Wilcoxon signed-rank test with FDR-BH correction ($p < 0.05$), excluding VC.

Method	Score (↑)	DSC (↑)	PPV (↑)	TPR (↑)	LFPR (↓)	LTPR (↑)	VC (↑)
Tiramisu (Zhang et al., 2019a)	0.720 ± 0.093*	0.621 ± 0.215*	0.714 ± 0.183*	0.623 ± 0.257*	0.444 ± 0.223*	0.737 ± 0.249*	0.921
ModDrop++ (Liu et al., 2022)	0.718 ± 0.094*	0.622 ± 0.215*	0.702 ± 0.187*	0.626 ± 0.252*	0.467 ± 0.215*	0.745 ± 0.239*	0.933
CD (Feng et al., 2019)	0.715 ± 0.095*	0.604 ± 0.221*	0.665 ± 0.198*	0.622 ± 0.261*	0.440 ± 0.215*	0.730 ± 0.247*	0.936
DG (BN) (Zhang et al., 2023a)	0.737 ± 0.082*	0.666 ± 0.189*	0.691 ± 0.189*	0.707 ± 0.205*	0.485 ± 0.217*	**0.812 ± 0.214***	0.943
DG (IN) (Zhang et al., 2023a)	0.749 ± 0.081*	0.680 ± 0.178*	0.702 ± 0.190*	**0.713 ± 0.193***	0.444 ± 0.219*	0.803 ± 0.209*	**0.945**
nnU-Net (Isensee et al., 2021)	0.777 ± 0.078	0.685 ± 0.180	0.739 ± 0.184*	0.698 ± 0.207*	0.242 ± 0.186	0.700 ± 0.248	0.939
WMH-SynthSeg (Laso et al., 2024)	0.467 ± 0.119*	0.280 ± 0.189*	0.347 ± 0.274*	0.287 ± 0.180*	0.394 ± 0.261*	0.282 ± 0.186*	0.665
UNISELF (Proposed)	**0.783 ± 0.074**	**0.686 ± 0.177**	**0.763 ± 0.165**	0.676 ± 0.205	**0.227 ± 0.177**	0.690 ± 0.251	0.944

(*: statistically significant difference compared to UNISELF (Proposed) in each column, based on paired Wilcoxon signed-rank test with FDR-BH correction ($p < 0.05$), excluding VC. The best and second-best performances in each column are shown in **bold** and underline, respectively.)

Table S6. Performance comparison of segmentation methods on the private test dataset (No FLAIR). "UNISELF (Proposed)" denotes the final configuration selected via cross-validation. Statistical significance assessed using paired Wilcoxon signed-rank test with FDR-BH correction ($p < 0.05$), excluding VC.

Method	Score (↑)	DSC (↑)	PPV (↑)	TPR (↑)	LFPR (↓)	LTPR (↑)	VC (↑)
Tiramisu (Zhang et al., 2019a)	0.656 ± 0.102*	0.458 ± 0.202*	0.727 ± 0.215*	0.383 ± 0.216*	0.388 ± 0.232*	0.553 ± 0.265*	0.866
ModDrop++ (Liu et al., 2022)	0.624 ± 0.108*	0.388 ± 0.238*	0.721 ± 0.242*	0.315 ± 0.229*	0.378 ± 0.235*	0.477 ± 0.271	0.841
CD (Feng et al., 2019)	0.559 ± 0.110*	0.185 ± 0.179*	0.699 ± 0.404	0.118 ± 0.129*	**0.063 ± 0.138***	0.220 ± 0.213*	0.638
DG (BN) (Zhang et al., 2023a)	0.606 ± 0.104*	0.364 ± 0.264*	0.644 ± 0.311*	0.314 ± 0.254*	0.275 ± 0.245*	0.391 ± 0.303*	0.804
DG (IN) (Zhang et al., 2023a)	0.676 ± 0.099*	**0.516 ± 0.196**	0.693 ± 0.194*	**0.471 ± 0.229***	0.432 ± 0.225*	**0.642 ± 0.246***	0.891
nnU-Net (Isensee et al., 2021)	0.690 ± 0.101*	0.515 ± 0.188	0.713 ± 0.187*	0.447 ± 0.205	0.214 ± 0.201*	0.466 ± 0.245*	0.895
WMH-SynthSeg (Laso et al., 2024)	0.453 ± 0.112*	0.253 ± 0.167*	0.352 ± 0.266*	0.239 ± 0.150*	0.476 ± 0.257*	0.252 ± 0.167*	0.732
UNISELF (Proposed)	**0.722 ± 0.086**	0.511 ± 0.212	**0.799 ± 0.177**	0.428 ± 0.230	0.154 ± 0.173	0.489 ± 0.271	**0.897**

(*: statistically significant difference compared to UNISELF (Proposed) in each column, based on paired Wilcoxon signed-rank test with FDR-BH correction ($p < 0.05$), excluding VC. The best and second-best performances in each column are shown in **bold** and underline, respectively.)

Table S7. Performance comparison of segmentation methods on the private test dataset (Motion Artifacts on FLAIR). "UNISELF (Proposed)" denotes the final configuration selected via cross-validation. Statistical significance asse ssed using paired Wilcoxon signed-rank test with FDR-BH correction ($p < 0.05$), excluding VC.

Method	Score (↑)	DSC (↑)	PPV (↑)	TPR (↑)	LFPR (↓)	LTPR (↑)	VC (↑)
Tiramisu (Zhang et al., 2019a)	0.683 ± 0.105*	0.579 ± 0.219*	0.657 ± 0.207*	0.596 ± 0.268*	0.457 ± 0.244*	0.674 ± 0.268*	0.896
ModDrop++ (Liu et al., 2022)	0.685 ± 0.106*	0.589 ± 0.214*	0.651 ± 0.190*	0.606 ± 0.253*	0.506 ± 0.222*	0.702 ± 0.253*	0.922
CD (Feng et al., 2019)	0.675 ± 0.110*	0.549 ± 0.229*	0.635 ± 0.204*	0.552 ± 0.272*	0.452 ± 0.213*	0.636 ± 0.267*	0.926
DG (BN) (Zhang et al., 2023a)	0.707 ± 0.093*	0.635 ± 0.191*	0.663 ± 0.187*	0.670 ± 0.214*	0.503 ± 0.214*	**0.746 ± 0.241***	0.938
DG (IN) (Zhang et al., 2023a)	0.715 ± 0.099*	0.650 ± 0.183*	0.670 ± 0.193*	**0.683 ± 0.202***	0.484 ± 0.223*	0.745 ± 0.233*	**0.940**
nnU-Net (Isensee et al., 2021)	0.753 ± 0.094*	**0.656 ± 0.184***	0.712 ± 0.181*	0.660 ± 0.213*	0.232 ± 0.186	0.629 ± 0.257*	0.931
WMH-SynthSeg (Laso et al., 2024)	0.455 ± 0.119*	0.277 ± 0.180*	0.346 ± 0.267*	0.287 ± 0.174*	0.407 ± 0.262*	0.268 ± 0.187*	0.648
UNISELF (Proposed)	**0.763 ± 0.096**	0.656 ± 0.185	**0.733 ± 0.175**	0.642 ± 0.214	**0.213 ± 0.189**	0.633 ± 0.265	0.936

(*: statistically significant difference compared to UNISELF (Proposed) in each column, based on paired Wilcoxon signed-rank test with FDR-BH correction ($p < 0.05$), excluding VC. The best and second-best performances in each column are shown in **bold** and underline, respectively.)

Table S8. Performance comparison of segmentation methods on the private test dataset (Noise on FLAIR). "UNISELF (Proposed)" denotes the final configuration selected via cross-validation. Statistical significance assessed using paired Wilcoxon signed-rank test with FDR-BH correction ($p < 0.05$), excluding VC.

Method	Score (↑)	DSC (↑)	PPV (↑)	TPR (↑)	LFPR (↓)	LTPR (↑)	VC (↑)
Tiramisu (Zhang et al., 2019a)	0.712 ± 0.092*	0.597 ± 0.221*	0.731 ± 0.175*	0.574 ± 0.259*	0.438 ± 0.228*	0.708 ± 0.258*	0.915
ModDrop++ (Liu et al., 2022)	0.716 ± 0.094*	0.612 ± 0.212*	0.731 ± 0.182*	0.581 ± 0.240*	0.459 ± 0.210*	0.719 ± 0.245*	0.934
CD (Feng et al., 2019)	0.717 ± 0.090*	0.593 ± 0.227*	0.707 ± 0.182*	0.580 ± 0.268*	0.414 ± 0.206*	0.697 ± 0.255*	0.934
DG (BN) (Zhang et al., 2023a)	0.732 ± 0.085*	0.659 ± 0.189	0.711 ± 0.183*	0.671 ± 0.207*	0.486 ± 0.213*	**0.789 ± 0.226***	0.942
DG (IN) (Zhang et al., 2023a)	0.745 ± 0.083*	0.675 ± 0.178	0.718 ± 0.186*	0.685 ± 0.194*	0.447 ± 0.216*	0.787 ± 0.216*	**0.945**
nnU-Net (Isensee et al., 2021)	0.774 ± 0.085	**0.682 ± 0.179**	0.733 ± 0.182*	**0.693 ± 0.205***	0.236 ± 0.183*	0.684 ± 0.258*	0.942
WMH-SynthSeg (Laso et al., 2024)	0.453 ± 0.120*	0.272 ± 0.177*	0.396 ± 0.285*	0.246 ± 0.161*	0.406 ± 0.271*	0.225 ± 0.162*	0.660
UNISELF (Proposed)	**0.779 ± 0.079**	0.671 ± 0.180	**0.773 ± 0.161**	0.641 ± 0.206	**0.208 ± 0.169**	0.661 ± 0.258	0.943

(*: statistically significant difference compared to UNISELF (Proposed) in each column, based on paired Wilcoxon signed-rank test with FDR-BH correction ($p < 0.05$), excluding VC. The best and second-best performances in each column are shown in **bold** and underline, respectively.)

Table S9. Performance comparison of segmentation methods on the private test dataset (Ghosting Artifacts on FLAIR). "UNISELF (Proposed)" denotes the final configuration selected via cross-validation. Statistical significance assessed using paired Wilcoxon signed-rank test with FDR-BH correction ($p < 0.05$), excluding VC.

Method	Score (↑)	DSC (↑)	PPV (↑)	TPR (↑)	LFPR (↓)	LTPR (↑)	VC (↑)
Tiramisu (Zhang et al., 2019a)	0.680 ± 0.093*	0.578 ± 0.212*	0.672 ± 0.185*	0.580 ± 0.254*	0.525 ± 0.216*	0.708 ± 0.251*	0.914
ModDrop++ (Liu et al., 2022)	0.684 ± 0.096*	0.587 ± 0.208*	0.665 ± 0.194*	0.590 ± 0.238*	0.543 ± 0.206*	0.724 ± 0.242*	0.928
CD (Feng et al., 2019)	0.676 ± 0.098*	0.546 ± 0.229*	0.633 ± 0.208*	0.549 ± 0.268*	0.477 ± 0.210*	0.663 ± 0.257*	0.928
DG (BN) (Zhang et al., 2023a)	0.706 ± 0.085*	0.632 ± 0.192*	0.667 ± 0.195*	0.661 ± 0.205*	0.549 ± 0.199*	**0.781 ± 0.226***	0.941
DG (IN) (Zhang et al., 2023a)	0.718 ± 0.089*	0.648 ± 0.180*	0.680 ± 0.194*	**0.668 ± 0.191***	0.503 ± 0.208*	0.768 ± 0.218*	**0.942**
nnU-Net (Isensee et al., 2021)	0.753 ± 0.092*	0.646 ± 0.183*	0.732 ± 0.190	0.629 ± 0.205	0.247 ± 0.189	0.638 ± 0.252*	0.933
WMH-SynthSeg (Laso et al., 2024)	0.422 ± 0.117*	0.260 ± 0.179*	0.328 ± 0.268*	0.270 ± 0.167*	0.475 ± 0.269*	0.259 ± 0.175*	0.612
UNISELF (Proposed)	**0.765 ± 0.083**	**0.656 ± 0.177**	**0.743 ± 0.172**	0.636 ± 0.201	**0.228 ± 0.176**	0.649 ± 0.250	0.940

(*: statistically significant difference compared to UNISELF (Proposed) in each column, based on paired Wilcoxon signed-rank test with FDR-BH correction ($p < 0.05$), excluding VC. The best and second-best performances in each column are shown in **bold** and underline, respectively.)

Table S10. Performance comparison of segmentation methods on the private test dataset (Bias Field Artifacts on FLAIR). "UNISELF (Proposed)" denotes the final configuration selected via cross-validation. Statistical significance assessed using paired Wilcoxon signed-rank test with FDR-BH correction ($p < 0.05$), excluding VC.

Method	Score (↑)	DSC (↑)	PPV (↑)	TPR (↑)	LFPR (↓)	LTPR (↑)	VC (↑)
Tiramisu (Zhang et al., 2019a)	0.711 ± 0.098*	0.586 ± 0.207*	0.701 ± 0.197*	0.557 ± 0.235	0.420 ± 0.222*	0.692 ± 0.255*	0.928
ModDrop++ (Liu et al., 2022)	0.718 ± 0.091*	0.598 ± 0.197*	0.738 ± 0.181*	0.550 ± 0.218*	0.402 ± 0.206*	0.679 ± 0.239*	0.925
CD (Feng et al., 2019)	0.673 ± 0.112*	0.535 ± 0.234*	0.596 ± 0.259*	0.555 ± 0.247	0.425 ± 0.215*	0.642 ± 0.249*	0.909
DG (BN) (Zhang et al., 2023a)	0.739 ± 0.088*	**0.656 ± 0.189***	0.715 ± 0.187*	**0.661 ± 0.204***	0.440 ± 0.216*	**0.768 ± 0.231***	0.944
DG (IN) (Zhang et al., 2023a)	0.746 ± 0.088*	0.646 ± 0.183*	0.727 ± 0.190*	0.621 ± 0.196*	0.409 ± 0.218*	0.759 ± 0.221*	**0.948**
nnU-Net (Isensee et al., 2021)	0.750 ± 0.093*	0.607 ± 0.192*	**0.804 ± 0.183***	0.530 ± 0.210*	**0.183 ± 0.166**	0.586 ± 0.250*	0.893
WMH-SynthSeg (Laso et al., 2024)	0.463 ± 0.120*	0.281 ± 0.188*	0.349 ± 0.274*	0.290 ± 0.180*	0.406 ± 0.266*	0.279 ± 0.183*	0.663
UNISELF (Proposed)	**0.773 ± 0.083**	0.642 ± 0.186	0.789 ± 0.160	0.583 ± 0.207	0.186 ± 0.155	0.619 ± 0.252	0.945

(*: statistically significant difference compared to UNISELF (Proposed) in each column, based on paired Wilcoxon signed-rank test with FDR-BH correction ($p < 0.05$), excluding VC. The best and second-best performances in each column are shown in **bold** and underline, respectively.)

Table S11. Performance comparison of segmentation methods on the private test dataset (Blurriness on FLAIR). "UNISELF (Proposed)" denotes the final configuration selected via cross-validation. Statistical significance assessed using paired Wilcoxon signed-rank test with FDR-BH correction ($p < 0.05$), excluding VC.

Method	Score (↑)	DSC (↑)	PPV (↑)	TPR (↑)	LFPR (↓)	LTPR (↑)	VC (↑)
Tiramisu (Zhang et al., 2019a)	0.707 ± 0.096*	0.605 ± 0.206*	0.618 ± 0.203*	0.680 ± 0.252	0.390 ± 0.238*	0.684 ± 0.262*	0.921
ModDrop++ (Liu et al., 2022)	0.706 ± 0.100*	0.602 ± 0.199*	0.596 ± 0.193*	0.682 ± 0.242*	0.407 ± 0.245*	0.702 ± 0.256*	0.929
CD (Feng et al., 2019)	0.704 ± 0.106*	0.587 ± 0.208*	0.586 ± 0.211*	0.662 ± 0.251	0.363 ± 0.235*	0.665 ± 0.265*	0.927
DG (BN) (Zhang et al., 2023a)	0.729 ± 0.089*	0.644 ± 0.188*	0.632 ± 0.184*	**0.727 ± 0.215***	0.403 ± 0.223*	**0.740 ± 0.250***	**0.940**
DG (IN) (Zhang et al., 2023a)	0.742 ± 0.092*	0.659 ± 0.184*	0.660 ± 0.198*	0.717 ± 0.207	0.349 ± 0.228*	0.723 ± 0.250*	0.933
nnU-Net (Isensee et al., 2021)	0.767 ± 0.084*	0.661 ± 0.183*	**0.706 ± 0.185**	0.684 ± 0.213*	0.193 ± 0.178*	0.641 ± 0.259	0.936
WMH-SynthSeg (Laso et al., 2024)	0.472 ± 0.125*	0.305 ± 0.191*	0.336 ± 0.252*	0.345 ± 0.186*	0.392 ± 0.271*	0.306 ± 0.191*	0.652
UNISELF (Proposed)	**0.775 ± 0.085**	**0.674 ± 0.181**	0.698 ± 0.180	0.708 ± 0.213	**0.161 ± 0.149**	0.643 ± 0.267	0.934

(*: statistically significant difference compared to UNISELF (Proposed) in each column, based on paired Wilcoxon signed-rank test with FDR-BH correction ($p < 0.05$), excluding VC. The best and second-best performances in each column are shown in **bold** and underline, respectively.)

Table S12. Performance comparison of segmentation methods on the private test dataset (Anisotropy on FLAIR). "UNISELF (Proposed)" denotes the final configuration selected via cross-validation. Statistical significance assessed using paired Wilcoxon signed-rank test with FDR-BH correction ($p < 0.05$), excluding VC.

Method	Score (↑)	DSC (↑)	PPV (↑)	TPR (↑)	LFPR (↓)	LTPR (↑)	VC (↑)
Tiramisu (Zhang et al., 2019a)	0.678 ± 0.101*	0.564 ± 0.214*	0.661 ± 0.194*	0.563 ± 0.256*	0.449 ± 0.223*	0.644 ± 0.255*	0.906
ModDrop++ (Liu et al., 2022)	0.669 ± 0.098*	0.564 ± 0.212*	0.640 ± 0.182*	0.565 ± 0.251*	0.507 ± 0.194*	0.661 ± 0.246*	0.922
CD (Feng et al., 2019)	0.683 ± 0.097*	0.550 ± 0.229*	0.633 ± 0.196*	0.553 ± 0.269*	0.415 ± 0.211*	0.625 ± 0.247*	0.933
DG (BN) (Zhang et al., 2023a)	0.707 ± 0.086*	0.627 ± 0.189	0.661 ± 0.184*	0.653 ± 0.209*	0.480 ± 0.207*	**0.725 ± 0.236***	0.940
DG (IN) (Zhang et al., 2023a)	0.719 ± 0.089*	0.641 ± 0.176	0.671 ± 0.186*	**0.664 ± 0.195***	0.442 ± 0.217*	0.721 ± 0.231*	**0.942**
nnU-Net (Isensee et al., 2021)	0.731 ± 0.093*	0.613 ± 0.187*	0.692 ± 0.185*	0.597 ± 0.210*	0.244 ± 0.181*	0.581 ± 0.241*	0.935
WMH-SynthSeg (Laso et al., 2024)	0.445 ± 0.114*	0.276 ± 0.184*	0.327 ± 0.259*	0.298 ± 0.177*	0.447 ± 0.246*	0.279 ± 0.181*	0.645
UNISELF (Proposed)	**0.758 ± 0.080**	**0.642 ± 0.177**	**0.722 ± 0.160**	0.627 ± 0.208	**0.202 ± 0.161**	0.611 ± 0.250	0.940

(*: statistically significant difference compared to UNISELF (Proposed) in each column, based on paired Wilcoxon signed-rank test with FDR-BH correction ($p < 0.05$), excluding VC. The best and second-best performances in each column are shown in **bold** and underline, respectively.)

Table S13. Impact of different TTIN variants (Section 3.3) on segmentation scores (Eq. (5)) comparing models trained with and without contrast dropout (CD). When CD was not applied, independent models were trained separately for each input contrast combination, where CondIN and IN are identical for independently trained models. All input multicontrast image combinations from the 2015 ISBI challenge test dataset were tested and compared. Self-ensembled lesion fusion (Section 3.2) was applied to all models with specific τ_1 and τ_2 values corresponding to each configuration, as indicated in the third row. Rows highlighted in teal and gold indicate input contrast combinations where the same CD-trained model shows a noticeable improvement when switching from BN training statistics to TTIN statistics at test-time.

Input contrasts				BN training stats		TTIN trained with BN		TTIN trained with IN		TTIN trained with CondIN	
T1w	T2w	PDw	FLAIR	without CD	with CD	without CD	with CD	without CD	with CD	without CD	with CD (Proposed)
				$\tau_1 / \tau_2 = 20 / 12$	$\tau_1 / \tau_2 = 20 / 12$	$\tau_1 / \tau_2 = 18 / 12$	$\tau_1 / \tau_2 = 16 / 8$	$\tau_1 / \tau_2 = 18 / 8$	$\tau_1 / \tau_2 = 16 / 8$	$\tau_1 / \tau_2 = 18 / 8$	$\tau_1 / \tau_2 = 16 / 7$
▲	▲	▲	▲	93.204 ± 7.351	93.134 ± 7.070	93.174 ± 8.383	93.078 ± 7.613	93.350 ± 6.583	93.271 ± 7.234	93.350 ± 6.583	93.286 ± 7.400
▲	▲	▲		92.532 ± 6.886	92.093 ± 7.142	92.483 ± 7.762	92.319 ± 7.286	92.620 ± 7.419	92.353 ± 7.435	92.620 ± 7.419	92.386 ± 7.238
▲	▲		▲	93.255 ± 6.764	93.039 ± 7.110	93.171 ± 7.774	93.112 ± 7.721	93.319 ± 6.958	93.129 ± 7.645	93.319 ± 6.958	93.172 ± 7.933
▲		▲	▲	93.121 ± 7.662	93.028 ± 7.541	93.086 ± 7.763	93.074 ± 7.801	93.184 ± 7.227	93.036 ± 7.743	93.184 ± 7.227	93.145 ± 7.686
	▲	▲	▲	93.253 ± 7.084	92.494 ± 8.448	93.017 ± 7.736	92.986 ± 7.782	93.179 ± 7.508	93.117 ± 7.300	93.179 ± 7.508	93.131 ± 7.554
▲	▲			91.608 ± 6.830	88.714 ± 5.230	91.694 ± 6.652	91.777 ± 7.056	91.800 ± 7.688	91.981 ± 6.924	91.800 ± 7.688	91.891 ± 7.326
▲		▲		92.035 ± 6.032	91.221 ± 7.098	92.209 ± 6.918	91.839 ± 7.862	92.248 ± 6.768	92.028 ± 7.169	92.248 ± 6.768	91.987 ± 7.001
▲			▲	93.114 ± 7.856	92.473 ± 7.642	93.099 ± 7.635	92.768 ± 8.439	93.089 ± 7.272	92.905 ± 7.980	93.089 ± 7.272	92.987 ± 7.993
	▲	▲		92.293 ± 7.539	91.820 ± 6.387	92.374 ± 8.312	91.914 ± 7.342	92.483 ± 7.919	92.307 ± 7.834	92.483 ± 7.919	92.414 ± 7.132
	▲		▲	93.249 ± 7.417	91.714 ± 7.675	93.048 ± 7.866	92.701 ± 8.387	93.137 ± 7.781	92.905 ± 7.871	93.137 ± 7.781	92.870 ± 8.187
		▲	▲	92.985 ± 7.978	90.739 ± 12.58	92.794 ± 8.260	92.609 ± 8.653	93.002 ± 8.124	92.866 ± 7.904	93.002 ± 8.124	92.867 ± 7.989
▲				91.117 ± 6.226	nan ± nan	91.078 ± 6.520	90.786 ± 6.645	91.264 ± 6.577	91.070 ± 6.674	91.264 ± 6.577	91.053 ± 6.614
	▲			90.420 ± 11.23	85.240 ± 39.69	91.408 ± 7.565	90.972 ± 5.896	91.470 ± 6.849	91.541 ± 6.958	91.470 ± 6.849	91.471 ± 7.307
		▲		90.494 ± 9.037	87.766 ± 16.06	91.494 ± 6.865	90.818 ± 7.920	91.628 ± 7.350	91.187 ± 7.402	91.628 ± 7.350	91.129 ± 7.551
			▲	92.256 ± 9.322	85.727 ± 38.16	92.089 ± 9.331	91.684 ± 10.81	92.230 ± 9.106	92.184 ± 9.340	92.230 ± 9.106	92.070 ± 9.465

Table S14. Impact of different TTIN variants (Section 3.3) on segmentation scores (Eq. (5)) comparing models trained with and without contrast dropout (CD). When CD was not applied, independent models were trained separately for each input contrast combination. Public and private test datasets considering both original and missing FLAIR multicontrast inputs were tested and compared. Self-ensemble (Section 3.2) were applied to all models.

Method		2016 MICCAI Dataset		UMCL Dataset		Private Dataset	
Norm Stats	CD	Original (T1+T2+PD+FLAIR)	No FLAIR (T1+T2+PD)	Original (T1+T2+FLAIR)	No FLAIR (T1+T2)	Original (mixed inputs)	No FLAIR
BN training stats	✗	0.746 ± 0.062*	0.711 ± 0.039	0.720 ± 0.098*	0.651 ± 0.088*	0.744 ± 0.099*	0.679 ± 0.092*
BN training stats	✓	**0.773 ± 0.067**	**0.723 ± 0.064**	0.731 ± 0.078*	0.548 ± 0.065*	0.732 ± 0.106*	0.644 ± 0.120*
TTIN trained with BN	✗	0.734 ± 0.063*	0.709 ± 0.067	0.724 ± 0.091*	0.649 ± 0.113*	0.758 ± 0.090*	0.692 ± 0.088*
TTIN trained with BN	✓	0.767 ± 0.062	0.711 ± 0.057	0.730 ± 0.081*	0.674 ± 0.065	**0.785 ± 0.078**	0.720 ± 0.094
TTIN trained with IN/CondIN	✗	0.753 ± 0.056	0.717 ± 0.065	0.737 ± 0.103	0.657 ± 0.097*	0.759 ± 0.086*	0.693 ± 0.097*
TTIN trained with IN	✓	0.769 ± 0.060	0.722 ± 0.058	**0.745 ± 0.090**	**0.676 ± 0.077**	0.781 ± 0.080	**0.726 ± 0.085**
TTIN trained with CondIN (Proposed)	✓	0.770 ± 0.057	0.715 ± 0.066	**0.745 ± 0.085**	0.668 ± 0.100	0.783 ± 0.074	0.722 ± 0.086

(*: statistically significant in the paired Wilcoxon signed-rank test with FDR-BH correction compared to 'TTIN trained with CondIN + CD (Proposed)' (last row in the table) in each column, p-value < 0.05. The best and second-best performances in each column are denoted in **bold** and underline, respectively.)

Table S15. Same segmentation score comparison as Table S14 on the private test dataset with various FLAIR artifacts.

Method		FLAIR Artifacts in Private Dataset					
**Norm Stats**	**CD**	**Motion**	**Noise**	**Ghosting**	**Bias Field**	**Spatial Blurriness**	**Anisotropy**
BN training stats	✗	0.720 ± 0.110*	0.740 ± 0.107*	0.726 ± 0.102*	0.729 ± 0.096*	0.733 ± 0.094*	0.701 ± 0.109*
BN training stats	✓	0.699 ± 0.112*	0.712 ± 0.113*	0.704 ± 0.111*	0.741 ± 0.090*	0.746 ± 0.090*	0.719 ± 0.101*
TTIN trained with BN	✗	0.735 ± 0.101*	0.756 ± 0.093*	0.734 ± 0.100*	0.727 ± 0.109*	0.741 ± 0.096*	0.721 ± 0.093*
TTIN trained with BN	✓	**0.765 ± 0.089**	**0.782 ± 0.080**	**0.767 ± 0.081**	**0.778 ± 0.083**	<u>0.772 ± 0.086</u>	**0.761 ± 0.083**
TTIN trained with IN/CondIN	✗	0.738 ± 0.092*	0.759 ± 0.087*	0.739 ± 0.087*	0.744 ± 0.094*	0.727 ± 0.103*	0.716 ± 0.093*
TTIN trained with IN	✓	**0.765 ± 0.094**	0.778 ± 0.083	**0.767 ± 0.085**	0.767 ± 0.087	0.770 ± 0.089	0.757 ± 0.085
TTIN trained with CondIN (Proposed)	✓	<u>0.763 ± 0.096</u>	<u>0.779 ± 0.079</u>	<u>0.765 ± 0.083</u>	<u>0.773 ± 0.083</u>	**0.775 ± 0.085**	<u>0.758 ± 0.080</u>

(*: statistically significant in the paired Wilcoxon signed-rank test with FDR-BH correction compared to ' TTIN trained with CondIN + CD (Proposed)' (last row in the table) in each column, p-value < 0.05. The best and second-best performances in each column are denoted in **bold** and <u>underline</u>, respectively.)
